# A mouse monoclonal antibody against influenza C virus attenuates acetaminophen-induced liver injury in mice

**DOI:** 10.1038/s41598-021-91251-x

**Published:** 2021-06-03

**Authors:** Yutaka Sasaki, Naoto Yoshino, Takako Okuwa, Takashi Odagiri, Takashi Satoh, Yasushi Muraki

**Affiliations:** 1 Division of Infectious Diseases and Immunology, Department of Microbiology, School of Medicine, Iwate Medical University, 1-1-1 Idaidori, Yahaba, Iwate 028-3694 Japan; 2 Department of Microbiology, Kanazawa Medical University School of Medicine, Ishikawa, Japan; 3 Department of Pathology, School of Medicine, Iwate Medical University, Iwate, Japan

**Keywords:** Immunology, Microbiology, Diseases, Molecular medicine

## Abstract

Molecular mimicry is one of the main processes for producing autoantibodies during infections. Although some autoantibodies are associated with autoimmune diseases, the functions of many autoantibodies remain unknown. Previously, we reported that S16, a mouse (BALB/c) monoclonal antibody against the hemagglutinin-esterase fusion glycoprotein of influenza C virus, recognizes host proteins in some species of animals, but we could not succeed in identifying the proteins. In the present study, we found that S16 cross-reacted with acetyl-CoA acyltransferase 2 (ACAA2), which is expressed in the livers of BALB/c mice. ACAA2 was released into the serum after acetaminophen (APAP) administration, and its serum level correlated with serum alanine aminotransferase (ALT) activity. Furthermore, we observed that S16 injected into mice with APAP-induced hepatic injury prompted the formation of an immune complex between S16 and ACAA2 in the serum. The levels of serum ALT (*p* < 0.01) and necrotic areas in the liver (*p* < 0.01) were reduced in the S16-injected mice. These results suggest that S16 may have a mitigation function in response to APAP-induced hepatotoxicity. This study shows the therapeutic function of an autoantibody and suggests that an antibody against extracellular ACAA2 might be a candidate for treating APAP-induced hepatic injury.

## Introduction

Microbial infection is one of the common triggers for autoantibody production. Structure and/or sequence similarity between the infectious agent and host antigen induces autoantibody production due to molecular mimicry^1,2^. In addition, the fact that antibodies do not necessarily show strict specificity for a single epitope or antigen owing to polyspecificity should be considered for autoantibody production^3,4^. For instance, Srinivasappa et al. reported that approximately 3.5% of monoclonal antibodies (mAbs) against 11 different viruses cross-react with host antigens^5^. Furthermore, autoantibodies are occasionally associated with autoimmune diseases as they bind to antigens of the host cells^6–8^.

Conversely, some autoantibodies have been reported to mitigate disorders induced via inflammation^9–11^. Autoantibodies against high-mobility group box 1 (HMGB1), a 30-kDa DNA-binding protein, have been shown to ameliorate diseases. HMGB1 is predominantly expressed in the nucleus and is involved in the regulation of transcription, DNA repair, and genome stability^12^. HMGB1 is released into the extracellular space during infection, cell damage, or inflammation^13–15^, and extracellular HMGB1 induces inflammatory cytokine expression in systemic tissues, functioning as damage-associated molecular patterns (DAMPs). Recently, an autoantibody against extracellular HMGB1 has been shown to reduce hepatic injury and to be associated with a favorable outcome of septic shock^9,10^.

Based on the above observations, autoantibodies have been proposed to perform opposite functions—to induce and mitigate of disorders. Numerous studies have provided evidence for the role of polyspecific antibodies in the innate defense system^16–18^. Although autoantibodies are detected in 23.6% of healthy subjects^19^, many autoantibodies are nonspecifically produced by different diseases^20^. Recent findings on the polyspecificity support the use of polyspecific antibodies in clinical applications^21^.

The influenza C virus (FluC) is the causative agent of upper respiratory tract infections in humans^22^. The hemagglutinin-esterase fusion (HEF) glycoprotein on the surface of the virion is a major virus antigen that induces the generation of neutralizing antibodies in infected individuals^23–26^. Previously, we generated a panel of 37 mouse mAbs against HEF glycoprotein of the influenza C/Ann Arbor/1/50 by immunizing BALB/c mice with 50 μg purified proteins^27,28^. One of the mAbs, S16, possesses a unique property; it recognizes not only a linear epitope on HEF but also proteins of various host cells, including those of human, monkey, canine, and chicken origin^28^. However, whether S16 recognizes mouse proteins is not known, although it has been demonstrated to recognize a linear epitope composed of six amino acids (F[NAT]EE[NYA]L, except FAEEAL and FTEEAL)^29,30^.

In the present study, we identified a mouse liver protein, acetyl-CoA acyltransferase 2 (ACAA2), that reacts with S16. We further demonstrated the therapeutic effect of S16 on BALB/c mice with acetaminophen (APAP)-induced liver injury. The results may contribute to understanding the potential role of a polyspecific autoantibody. Furthermore, our data suggest that ACAA2 is a novel therapeutic target for drug-induced liver injury.

## Results

### Reactivity of S16 with proteins in cultured mouse cells

**Figure 1:**
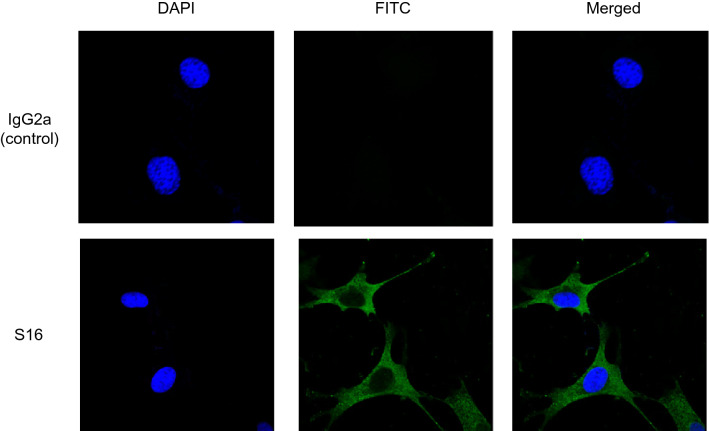
Immunofluorescence images of mouse NIH3T3 cells stained for S16. Uninfected mouse NIH3T3 cells were fixed with 4% paraformaldehyde and permeabilized with cold methanol. An immunofluorescence assay was performed using FITC-conjugated IgG2a isotype control (upper panels) or FITC-conjugated S16 (lower panels). The cells were observed for DAPI (left panels) or FITC (middle panels). The merged images are shown (right panels).

S16 recognizes not only the HEF protein of FluC but also proteins of uninfected cells derived from humans, monkeys, dogs, and chickens^28^. To assess whether S16 also recognizes self-antigens, we performed an immunofluorescence assay using a mouse fibroblast cell line (NIH3T3 cells). Fluorescent signals were not detected in cells stained with fluorescein isothiocyanate (FITC)-conjugated IgG2a (IgG2a-FITC) (Fig. 1, upper panels). In contrast, a clear fluorescent signal was detected in cells stained with FITC-conjugated S16 (S16-FITC) (Fig. 1, lower panels). In the merged image, the fluorescence of S16 was distributed throughout the cell, except in the nucleus. As shown in Supplementary Fig. S1, mouse L929 cells were also clearly stained with S16 in the same localization pattern. These results indicated that S16 is an autoantibody, as it reacts with mouse protein.

### Distribution of S16-binding proteins in mouse organs

**Figure 2:**
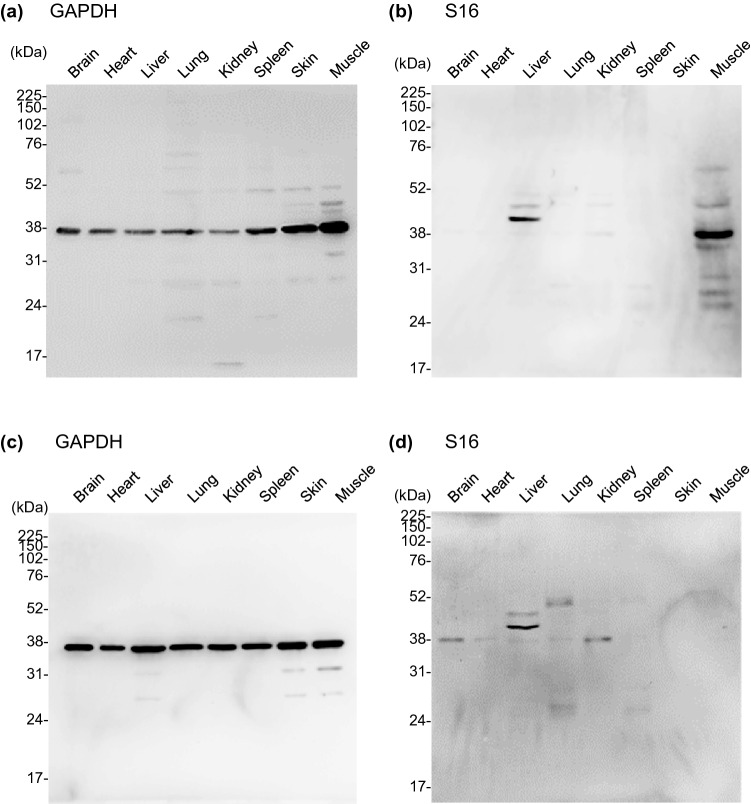
Organ distribution of S16-binding protein. Organs (brain, heart, liver, lung, kidney, spleen, skin, and muscle) were harvested from two different BALB/c mice (7-week-old, female). Upper and lower images refer to individual mice: mouse 1 (**a,b**) and mouse 2 (**c,d**). The proteins extracted from the organs (3 μg) were separated on a 12% SDS-PAGE, followed by western blotting with α-mouse GAPDH mAb (**a,c**) or S16 (**b,d**). Molecular weight markers are shown at the left of the gels (kDa).

To identify mouse proteins that react with S16, we analyzed the lysates of NIH3T3 and L929 cells using western blotting (Supplementary Figure S2). As multiple bands were detected, it was unlikely that specific proteins could be identified. Therefore, we attempted to gain specificity by analyzing proteins from mouse organ extracts. We examined the expression of S16-binding proteins and their distribution in organs from BALB/c mice organs via western blotting (Fig. 2 and Supplementary Figures S3, S4). Proteins extracted from the organs (brain, heart, liver, lung, kidney, spleen, skin, or muscle) of five different BALB/c mice were probed using rabbit α-mouse GAPDH or S16 as the primary antibodies. GAPDH was detected in all organs, and the expression levels were virtually identical, with slightly higher levels in the skin and muscle (Fig. 2a,c). In one mouse, S16-binding proteins of ~ 40 kDa molecular weight (MW) were detected in the liver and muscle, respectively (Fig. 2b). In the other mouse, although some weak bands were detected in several organs, a single clear band, the electrophoretic mobility of which was identical to that of the first mouse, was observed in the liver (Fig. 2d). Similar results were obtained in the other three mice (Supplementary Figure S3, S4). Taken together, these data strongly suggested that S16 binds stably to a liver protein of ~ 40 kDa.

### Subcellular localization of the S16-binding protein

**Figure 3:**
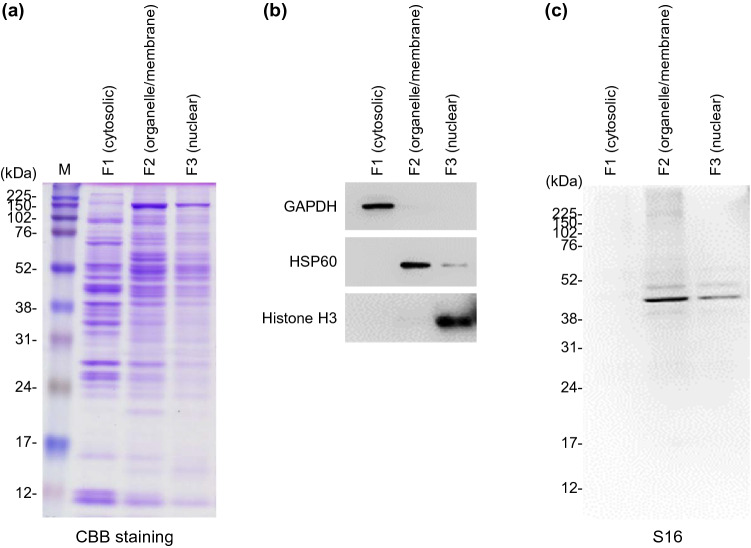
Subcellular localization of S16-binding protein in liver. Homogenized livers of BALB/c mice (7-week-old, female, n = 3) were fractionated into the cytosolic (F1), organelle/membrane (F2), and nuclear (F3) fractions. Proteins (3 μg) were separated on a 12% SDS-PAGE with reference molecular weight markers (M). Gels were subjected to CBB staining (**a**) or western blotting using α-mouse GAPDH mAb, α-mouse HSP60 mAb, α-mouse histone H3 mAb (**b**), or S16 (**c**). Molecular weight markers are shown at the left of the gels (kDa). Images shown are the representative results of three independent experiments.

Next, to examine the subcellular localization of the S16-binding protein, we analyzed the subcellular fractions of mouse livers. The obtained fractions (the cytosol, organelle/membrane, and nucleus) were subjected to sodium dodecyl sulfate–polyacrylamide gel electrophoresis (SDS-PAGE) followed by western blotting using rabbit α-mouse GAPDH, HSP60, or histone H3 as the primary antibodies (Fig. 3 and Supplementary Figure S5). In the gel stained with Coomassie Brilliant Blue (CBB), we confirmed that a specific amount of protein was recovered in each fraction without apparent degradation (Fig. 3a and Supplementary Figure S5). GAPDH (a cytosolic marker) and histone H3 (a nuclear marker) were detected only in F1 (the cytosolic fraction) and F3 (the nuclear fraction), respectively. HSP60 (a mitochondrial marker) was primarily detected in F2 (the organelle/membrane fraction), although trace amounts were detected in F3 (Fig. 3b and Supplementary Figure S5). After probing immunoblots with S16, we observed that the S16-binding protein (~ 40 kDa) was primarily expressed in F2 but not in F1 (Fig. 3c and Supplementary Figure S5). These results suggest that S16 primarily reacts with a protein recovered in the organelle/membrane fraction but not in the cytosolic fraction of the mouse liver.

### Immunoprecipitation and mass spectrometry (MS) analysis of the S16-binding protein

**Figure 4:**
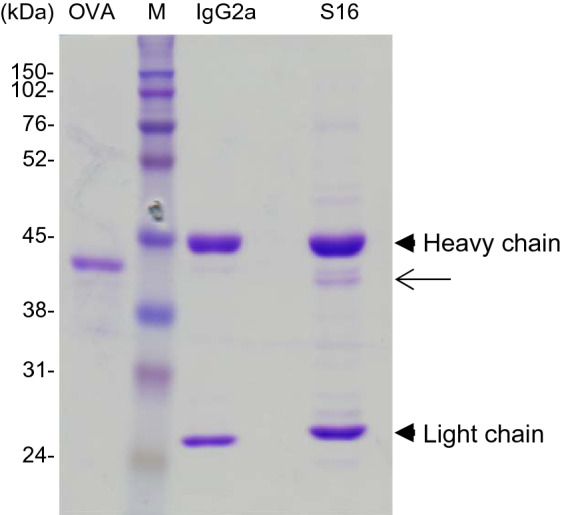
Immunoprecipitation of S16-binding protein. Liver proteins (200 μg) were immunoprecipitated with IgG2a isotype control or S16 using protein A dynabeads at room temperature for 15 min. The immunoprecipitated proteins and ovalbumin (OVA, 44.3 kDa) were electrophoresed using SDS-PAGE on 12% gels and visualized using CBB staining. The band of interest (arrow) was cut and subjected to MS analysis. Molecular weight markers (M) are separated, and the sizes are shown at the left of the gels (kDa). The image is the representative result of three independent experiments.

As S16 mainly reacts with the organelle/membrane fraction of the mouse liver (Fig. 3), we attempted to identify the S16-binding protein using MS analysis with the liver lysates. The lysate immunoprecipitated with either IgG2a or S16 was analyzed using SDS-PAGE (Fig. 4). Minor bands were detected in the IgG2a and S16 lanes as low background. A protein detected at approximately 40 kDa co-immunoprecipitated with S16 (Fig. 4 (arrow) and Supplementary Figure S6). As the MW of ovalbumin (OVA) is well known at 44.3 kDa, the MW of the immunoprecipitated protein was estimated to be between 38 and 45 kDa. Based on the identification scores of unique peptides, 45 proteins were detected in the MS analysis using an in-gel digestion method (Supplementary Tables S1 and S2). In addition, we analyzed S16-binding protein candidates using an on-bead digestion method (Supplementary Tables S1 and S3). A total of 207 proteins were identified via MS analysis and the top 45 proteins are listed in Supplementary Table S3.

### Confirming the binding between S16 and ACAA2

**Figure 5:**
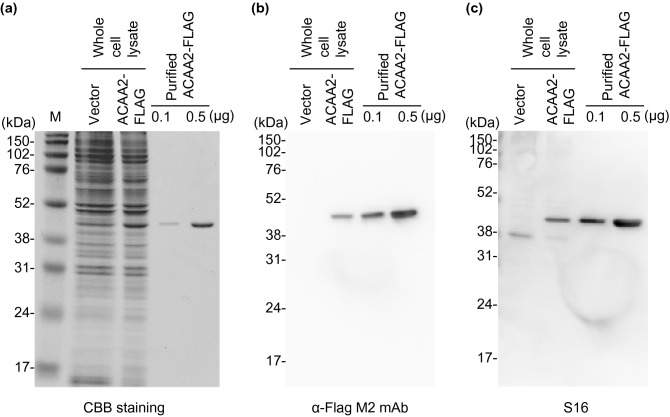
Binding of S16 with ACAA2. HEK293 cells were transfected with vector alone or ACAA2-FLAG expressing vector. The ACAA2-FLAG expressed in the cells was purified. The whole cell lysate (2 μg) transfected with vector alone (Vector), ACAA2-FLAG, and purified ACAA2-Flag (0.1 and 0.5 μg) were separated using 12% SDS-PAGE. The gel was subjected to CBB staining (**a**) and western blotting using α-Flag M2 mAb (**b**) or S16 (**c**). Molecular weight markers are shown at the left of the gels (kDa). The images shown are representative results of three independent experiments.

In MS analysis, considering the high number of unique peptides (Supplementary Tables S2 and S3), ACAA2 (acetyl-CoA acyltransferase 2, an alternative name of 3-ketoacyl-CoA thiolase) was identified as the first and second possible candidates from both in-gel and on-bead digestion methods, respectively. Taking the MW (41.8 kDa) and its localization in the organelle/membrane fraction (Supplementary Figure S7) into account, we considered that ACAA2 was the protein most likely recognized by S16. To confirm that S16 reacted to ACAA2, we performed western blotting of a FLAG-conjugated recombinant mouse ACAA2 (ACAA2-FLAG) protein using α-FLAG M2 mAb and S16 as the primary antibodies (Fig. 5 and Supplementary Figure S8). Mouse ACAA2-FLAG was purified using FLAG-affinity agarose beads, and the purity was confirmed using CBB staining (Fig. 5a and Supplementary Figure S8). A single band of approximately 40 kDa was observed in the lanes with purified ACAA2-FLAG (0.1 and 0.5 μg). The expression of ACAA2-FLAG was confirmed using western blotting with the α-FLAG M2 mAb (Fig. 5b and Supplementary Figure S8). The binding of S16 with ACAA2-FLAG was detected in the transfected cell lysate and purified ACAA2-FLAG; however, the binding was negligible in the vector-transfected cells (Vector) (Fig. 5c and Supplementary Figure S8). These results indicate that S16 binds to mouse ACAA2.

### Establishment of a mouse model of APAP-induced hepatic injury

**Figure 6:**
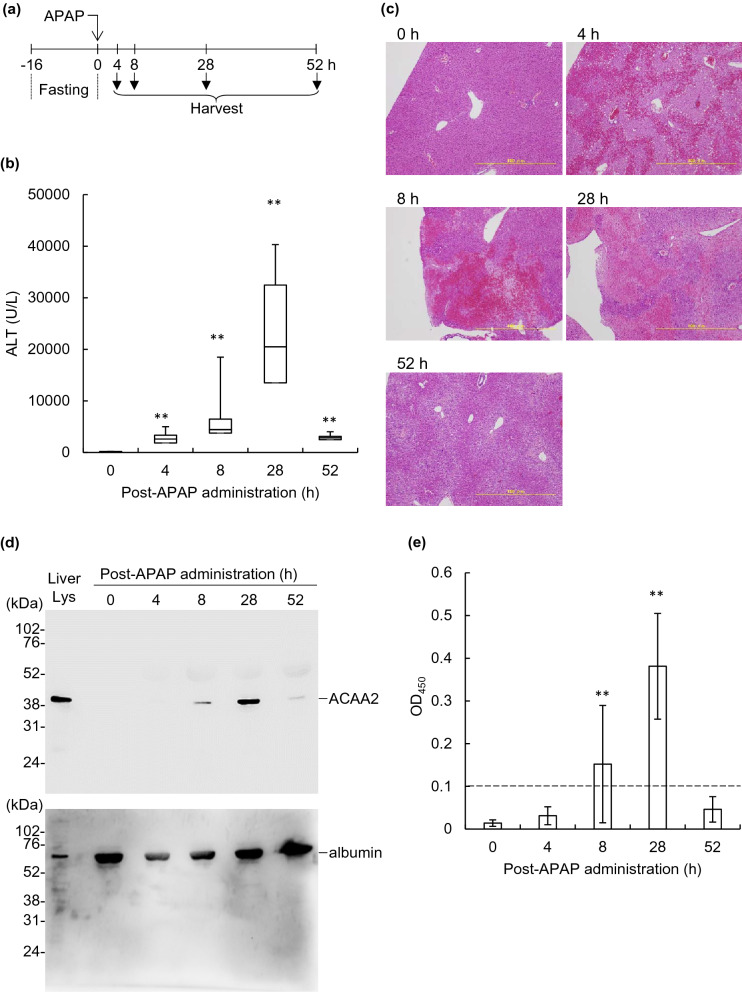
Establishment of a mouse model with APAP-induced hepatic injury. (**a**) Schematic diagram of the experiment. BALB/c mice (n = 10, at each time point) were fasted for 16 h. At 0 h, the mice were intraperitoneally administered APAP, and sacrificed at 4, 8, 28, and 52 h for sample collection. (**b**) Serum ALT levels were quantified using Fuji Dri-Chem NX 500v. The levels are expressed as median values with the interquartile range from more than three independent experiments. The significant differences between APAP from untreated and treated samples are indicated using asterisks (**p < 0.01). (**c**) Hepatic histology (H&E staining) at 0, 4, 8, 28, and 52 h post APAP-administration are shown. Images are representative of the treated mice. Scale bar = 100 μm. (**d**) Serum ACAA2 (upper panel) and albumin (lower panel) were detected using western blotting with α-mouse ACAA2 mAb and α-mouse albumin pAb, respectively. Liver lysate sample (3 μg) was used as the control. (**e**) The serum levels of ACAA2 were determined using ELISA. The serum ACAA2 level represents the mean ± SD from more than three independent experiments. The significant differences between APAP from untreated and treated samples are indicated using asterisks (**p < 0.01). The dotted line (OD_450_ = 0.1) indicates the detection limit.

Mouse ACAA2 is expressed in the mitochondria of hepatocytes^31^. The hepatic ACAA2 is released from the intracellular space into the serum because of APAP-induced hepatotoxicity^32^, whereas the function of extracellular ACAA2 is not fully understood. To assess the serum levels of ACAA2 in APAP-treated mice, western blotting and an enzyme-linked immunosorbent assay (ELISA) were performed at 0, 4, 8, 28, and 52 h after APAP was administered (Fig. 6a). First, APAP-induced hepatotoxicity was confirmed by quantifying serum ALT levels (Fig. 6b). Base levels of ALT were detected in the serum of non-APAP-administered mice (120 U/L). A significant increase in the serum ALT level was observed 4 h after APAP administration, which peaked at 28 h (2,605, 4,450, and 20,500 U/L at 4, 8, and 28 h, respectively). Additionally, serum ALT levels decreased considerably at 52 h (2,840 U/L). Histological changes in the liver, as indicated with hematoxylin and eosin (H&E) staining, also corresponded to the changes in serum ALT levels (Fig. 6c); necrotic area around the centrilobular areas^33,34^ was prominently observed at 28 h post-APAP administration.

Next, we assessed the levels of serum ACAA2 (Fig. 6d, e, and Supplementary Figure S9). In our western blotting analyses, the band of serum albumin was detected in all lanes, with slight variations in each lane (Fig. 6d (lower panel) and Supplementary Figure S9 (right panels)). In contrast, an apparent increase and decrease in serum ACAA2 was observed (Fig. 6d (upper panel) and Supplementary Figure S9 (left panels)). Although the ACAA2 band was not detected in the serum of non-APAP-administered mice, it appeared 8 h after APAP administration. The band intensity peaked at 28 h and then decreased at 52 h. These results were confirmed using ELISA (Fig. 6e). The absorbance of sera at 450 nm at 0 and 4 h after APAP administration was lower than the cut-off value of 0.1. The absorbance increased after 8 h and peaked at 28 h (OD_450_ values were 0.152 and 0.381, respectively). Then, the absorbance returned to a level lower than the cut-off value at 52 h. Thus, the correlation between ALT and ACAA2 in the serum of APAP-treated mice suggests that the release of ACAA2 into the serum depends on the extent of hepatic injury.

### Immune complex of S16 with ACAA2 in mouse serum

**Figure 7:**
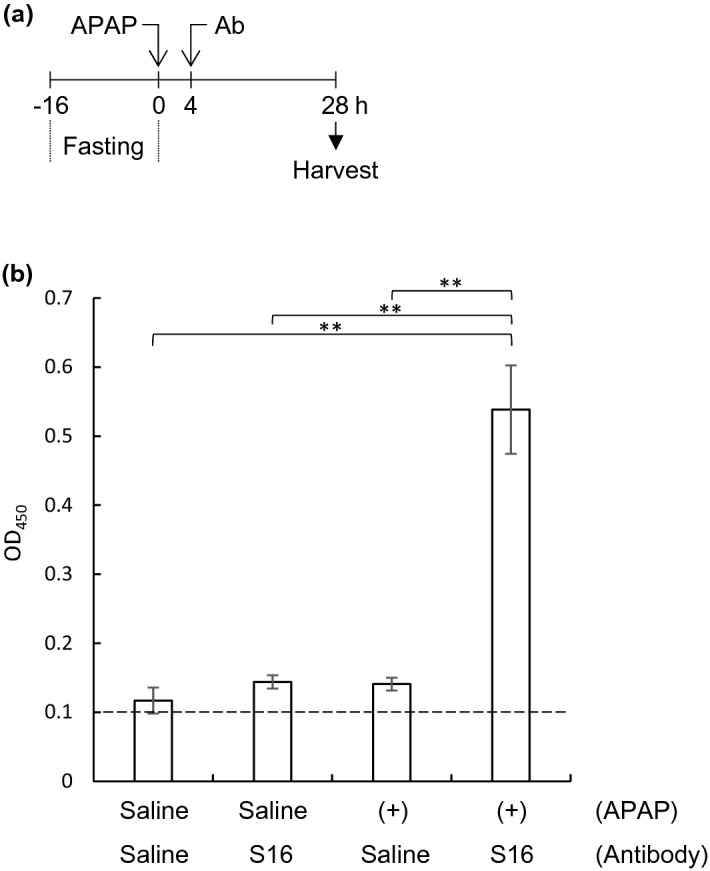
Complex formation between S16 and serum ACAA2. (**a**) Schematic diagram of the experiment. BALB/c mice (n = 6, in each group) were fasted for 16 h. At 0 h, the mice were intraperitoneally administered saline or APAP (300 mg/kg). At 4 h, the mice were injected intraperitoneally with saline or S16 (100 μg), and then sacrificed at 28 h. (**b**) Serum was collected from mice after 28 h of APAP administration, and complexes of S16 with ACAA2 in each serum sample were determined using sandwich ELISA. The values represent the mean ± SD from three independent experiments. Significant differences between groups are indicated using asterisks (**p < 0.01). The dotted line (OD_450_ = 0.1) indicates the detection limit.

Based on the results shown in Figs. 5 and 6, we hypothesized that S16 binds to ACAA2 in the serum of APAP-administered mice. To assess the possibility that the complex between S16 and ACAA2 is formed in the sera of mice, saline-, or APAP-administered mice were further injected with saline or S16 at 4 h and sacrificed at 28 h (Fig. 7a). Then, the complex formation between S16 and ACAA2 in the serum was analyzed using modified sandwich ELISA (Fig. 7b). In saline-administered mice, the absorbance at 450 nm was almost the same as the cut-off value of 0.1; the OD_450_ values of saline- and S16-injected mice were 0.117 and 0.144, respectively. A negligible change in absorbance was observed in the APAP-administered mice injected with saline (OD_450_ = 0.141). In contrast, compared to other mice, a significant increase in absorbance was detected in the APAP-treated mice injected with S16 (OD_450_ = 0.538). These findings suggest that S16 forms a complex with ACAA2 in the sera of APAP-treated mice.

### Effect of S16 on APAP-induced hepatotoxicity in mice

**Figure 8:**
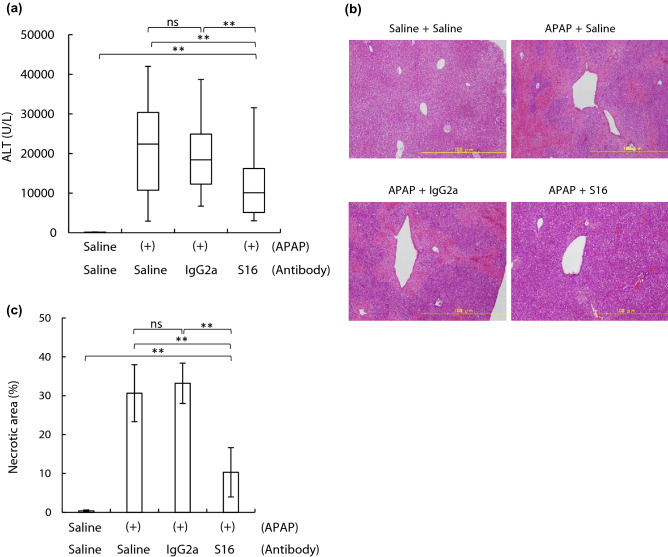
Effect of S16 on APAP-induced hepatotoxicity in mice. APAP (300 mg/kg) was intraperitoneally administered to BALB/c mice (7-week-old, female, n = 13, in each group). After 4 h of APAP treatment, the mice were injected with saline, IgG2a (100 μg), or S16 (100 μg). (**a**) ALT was quantified in the serum of APAP-administered mice after 28 h. ALT levels are expressed as median values with interquartile ranges from three independent experiments. (**b**) Hepatic histology following APAP administration were examined using H&E staining. The livers of normal mice (upper left) and APAP-administered mice injected with saline (upper right), IgG2a (lower left), or S16 (lower right) were harvested from five different mice. Images are representative of each treated mouse. Scale bar = 100 μm. (**c**) Necrotic areas in each H&E-stained section (shown in **b**) was quantified using the BZ-X analyzer. Data of the necrotic area are shown using the mean ± SD from five mice. In (**a**) and (**c**), significant differences between the S16 group and other groups are indicated using asterisks (**p < 0.01). ns, no significant differences between the saline group and the IgG2a group.

To investigate whether S16 affects APAP-induced hepatic injury, we evaluated biochemical and histological changes in APAP-administered mice with or without S16 injection (Fig. 8). Compared to those of the normal group (100 U/L), the serum ALT levels in the saline (22,400 U/L) and IgG2a groups (18,400 U/L) increased significantly, by 224- and 184-fold, respectively (Fig. 8a). Compared to that in normal mice, the serum ALT level also increased by 101-fold in mice injected with S16 (10,100 U/L). There was no significant difference in serum ALT levels between the saline and IgG2a groups. However, it is noteworthy that the serum ALT level in the S16 group was significantly lower than that in the saline group (55% decrease) or IgG2a group (45% decrease).

We next examined the hepatic histopathology of mice. In APAP-administered saline-injected mice, hepatocellular necrosis was observed around the centrilobular areas, a prominent feature of APAP-induced hepatotoxicity^33,34^ (Fig. 8b, upper right panel). As shown in Fig. 8c, quantification using the BZ-X analyzer revealed that the necrotic area accounted for 30.6% of the whole section, the value of which was significantly higher than that of normal mice (0.39%). Although the necrotic area was 33.2% in the IgG2a group, a significant reduction was observed in the S16 group (10.3%) compared to that in the saline or IgG2a group (Fig. 8b,c). Thus, based on biochemical and histological analysis, these data demonstrate the therapeutic effect of S16 on APAP-induced hepatotoxicity in mice.

## Discussion

This study demonstrated that S16, a mouse monoclonal α-HEF antibody, cross-reacts with mouse ACAA2. ACAA2 was released from the intracellular space into the serum upon APAP administration, and S16 injection led to the formation of immune complexes with serum ACAA2. We also found that injecting S16 into hepatic injured mice reduced the injury. Thus, we showed a potential mitigation function of S16 against APAP-induced hepatotoxicity and a promising therapeutic target for liver dysfunction.

We revealed that S16 binds to proteins in murine cell lines (i.e., NIH3T3 and L929 cells) and liver tissues (Figs. 1, 2, and Supplementary Figure S1), indicating that S16 is an autoantibody. Previously, we attempted to identify S16-binding proteins using cultured mouse cells, such as NIH3T3 and L929 cells, using western blotting. However, proteins were difficult to identify owing to the number of detected bands (Supplementary Figure S2); notably, previous reports have found similar results in which a monoclonal autoantibody bound not only to target proteins but also to other proteins^35,36^. In contrast, in the present study, we demonstrated that S16 consistently bound to a mouse liver protein (Fig. 2 and Supplementary Figure S3). Similar results were obtained using ELISA (Supplementary Figure S10). Interestingly, S16 highly reacted with a liver protein rather than other proteins in other organs. Thus, S16 is considered to be an autoantibody that recognizes a mouse protein. Since S16 consistently bound to a liver protein, we used mouse liver extracts in subsequent experiments.

In MS/MS analysis, we found more than 40 proteins were identified using the in-gel and on-bead digestion methods (Supplementary Table S1). In addition, we found ACAA2 was expressed in the organelle/membrane fraction (F2) of mouse liver extracts, which was also the fraction containing the S16-binding protein (Fig. 3 and Supplementary Fig. S7). Together with these data, ACAA2 was indicated to be a probable S16-binding protein, especially as it was the first and second protein with the most unique peptides identified via MS analysis (Supplementary Table S2 and S3). Indeed, we confirmed that S16 reacted with purified ACAA2-Flag (Fig. 5). Furthermore, we also found that ACAA2 co-immunoprecipitated with S16 (Supplementary Figure S11). These results clearly indicate that S16 binds to ACAA2.

Mouse ACAA2 has an S16 epitope-like sequence (199-FNEEMA-204), which is highly similar to the S16 epitope sequence^29,30^, but it differs at L204A. As the amino acids L and A have similar characteristics, the binding of mouse ACAA2 may also occur via this epitope. To support this hypothesis, the S16 epitope-like sequence (199-FNDEMA-204) in human ACAA2 (which shares 88% similarity with mouse ACAA2) was visualized on the 3D surface of human ACAA2 (Supplementary Figure S12). The structure of mouse ACAA2 has not been solved. Analysis of the epitope-like sequences in a manner similar as done by Okuwa et al.^30^ would be useful to understand the molecular basis for the cross-reactivity.

Autoantibodies against proteins released from hepatocytes are involved in the exacerbation or amelioration of disorders. Formiminotransferase cyclodeaminase (FTCD) is released into the serum from the Golgi apparatus of mouse hepatocytes upon APAP administration^32^. Autoantibodies against FTCD are related to the exacerbation of autoimmune hepatitis^37,38^. The autoantibody against HMGB1 mediates the reduction of hepatic injury and promotes a favorable outcome against septic shock^9,10^. Recently, Sun et al. showed that ACAA2 is released into the serum of C57/BL6 mice administered APAP^32^. In this study, we found that the release of ACAA2 into serum was detected at 8 h and reached a maximum level at 28 h after APAP treatment (Fig. 6 and Supplementary Figure S9). The kinetics of ACAA2 accumulation in serum were similar to the observed increases in of serum ALT levels. Therefore, these findings suggest that the prompt release of ACAA2 into serum following APAP administration may be correlated with hepatic function.

Previously, we administered S16 subcutaneously to BALB/c mice weekly for approximately 6 months to examine whether S16 induced disorders based on molecular mimicry (Supplementary Figure S13). The mice did not show any change; the body weight and hematological parameters (blood cell count) did not change in S16-injected mice compared to those in control mice. Furthermore, liver function was not affected by injecting S16 into normal mice, particularly as the expression level of miR-122, a microRNA marker for liver dysfunction, did not differ between IgG2a- and S16-injected mice (Supplementary Figure S14). These results indicate that S16 is non-pathogenic autoantibody rather than one that promotes autoimmune disease.

In contrast, S16 injected into mice with hepatic injury reduced APAP-induced hepatotoxicity. The necrosis area, which is the main effect from APAP-induced hepatotoxicity^39^, was reduced following S16 injection (Fig. 8b,c). Furthermore, an increase in absorbance at 450 nm was observed only in S16-injected hepatic injured mice but not in mice treated with saline or IgG2a, signifying that S16 forms an immune complex with extracellular ACAA2 without binding to intrinsic mouse antibodies (Fig. 7 and Supplementary Figure S15). These results suggest that complexes between S16 and extracellular ACAA2 associate with the mitigation of APAP-induced hepatotoxicity. In our preliminary experiments, the APAP-induced hepatic injury in mice was also mitigated by the injection of mouse α-human ACAA2 mAb (an antibody that cross-reacts with mouse ACAA2^40^); notably, a reduction in the serum ALT level was observed at 28 h in two out of the three mice examined (serum ALT levels at 28 h in the three mice were 21,200, 8900, and 9,700 U/L, compared to those in the saline group (mean ± SD; 15,350 ± 4653 U/L)). While further examination is needed whether other mouse proteins bind with S16 owing to polyspecificity and their contributions to attenuation of hepatic injury, our data support a notion that potential association of S16 with ACAA2 leads to the mitigation of hepatic injury.

In conclusion, S16, a mouse mAb against the FluC HEF protein, has polyspecificity and primarily reacts with the autoantigen, ACAA2. S16 forms a complex with extracellular ACAA2 and reduces hepatic injury in APAP-administered mice. Since S16 injection alone did not induce any disorders in BALB/c mice, the attenuation effect for disorders has to be considered during the functional analysis of autoantibodies that are not disease-related. For the first time, our study reveals the therapeutic function of non-pathogenic autoantibodies against the FluC HEF protein. Although there are still many questions regarding the mechanism of the observed phenomenon, the present study highlights the potential role of a polyspecific autoantibody that may be a promising tool for treating liver dysfunction.

## Methods

### Cells

Mouse fibroblast NIH3T3 cells (kindly provided by Dr. Toshiki Himeda, Kanazawa Medical University, Japan) and human embryonic kidney (HEK) 293 cells were cultured in Dulbecco’s modified Eagle’s medium (Invitrogen, Carlsbad, CA, USA) with 10% fetal bovine serum (Invitrogen) at 37 °C in the presence of 5% CO_2_. The HEK293 cells (JCRB9068, Lot 061496) were purchased from the National Institute of Health Sciences (Kanagawa, Japan).

### Antibodies

The mouse mAb S16 was produced by Sugawara et al.^27^. BALB/c mice were immunized with 50 μg purified HEF proteins of C/Ann Arbor/1/50 in Freund’s complete adjuvant thrice at weekly intervals. The hybridoma cells secreting antibodies to HEF were screened using enzyme-linked immunosorbent assay (ELISA). The isotype of S16 was classified as IgG2a using double immunodiffusion.

For western blotting, α-mouse ACAA2 (ab128911; Abcam, Cambridge, UK), α-GAPDH (#5174; Cell Signaling Technology, Danvers, MA, USA), α-HSP60 (#12165; Cell Signaling Technology), α-histone H3 (#4499; Cell Signaling Technology), α-Flag M2 (F1804; Sigma-Aldrich, St. Louis, MO, USA), and α-mouse albumin (#4929; Cell Signaling Technology) polyclonal antibodies (pAbs) were used as the primary antibodies. Horseradish peroxidase (HRP)-conjugated secondary antibodies against mouse and rabbit IgG were purchased from Bio-Rad (Hercules, CA, USA). The pAb against mouse ACAA2 (TA321746; OriGene, Rockville, MD, USA) and HRP-conjugated goat α-mouse IgG2a antibody (Southern Biotech, Birmingham, AL, USA) were used for ELISA. The IgG2a isotype control (BioLegend, San Diego, CA, USA) was used as a control antibody for the indirect immunofluorescence assay and injection of S16 into mice.

### Immunofluorescence assay

IgG2a isotype control and S16 were coupled with fluorescein isothiocyanate (FITC) using an FITC labeling kit (American Qualex International, Inc., La Mirada, CA, USA) according to the manufacturer’s protocol (IgG2a-FITC and S16-FITC, respectively). NIH3T3 cells (3.5 × 10^4^ cells/well) were cultured on glass coverslips in 12-well plates at 37 °C for 1 day. The cells were fixed with 4% paraformaldehyde for 20 min at room temperature and then permeabilized with cold methanol for 10 min at − 20 °C. The cells were washed thrice with phosphate-buffered saline (PBS) and incubated with 1:50 dilution of IgG2a-FITC or S16-FITC for 1 h at room temperature. The cells were then washed with PBS and stained with 4’,6-diamidino-2-phenylindole (DAPI) for 5 min. The samples were observed under a confocal laser scanning microscope (C1si; Nikon, Tokyo, Japan). Images were acquired using independent fluorescence channels at excitation wavelengths of 405 nm (DAPI) and 488 nm (FITC).

### Animals

Six-week-old female BALB/c mice were purchased from CLEA Japan, Inc., and housed in groups of five per plastic cage (225 × 338 × 140 mm^3^). Mice were maintained in a vivarium at 22  ± 1 °C under a 12-h light/dark cycle. The mice had ad libitum access to food and water under specific pathogen-free conditions for 1 week before being used in the experiments. Seven-week-old mice were used for each experiment. We have followed the ARRIVE guidelines regarding animal studies.

### Extraction and quantification of proteins

Organs (brain, heart, liver, lung, kidney, spleen, skin, and muscle) were harvested from five mice, and each organ was homogenized using gentleMACS (Miltenyi Biotec, Bergisch Gladbach, Germany). Proteins were extracted using radioimmunoprecipitation assay buffer, and protein concentration was quantified using the detergent compatible (DC) protein assay (Bio-Rad). In the text, the amount of protein is mentioned in parentheses.

### Western blotting

Sodium dodecyl sulfate–polyacrylamide gel electrophoresis (SDS-PAGE) and western blotting were performed as described previously^41^. Samples for analysis were separated using 12% SDS-PAGE under reducing conditions with a molecular weight marker (RPN800E, Amersham, Saclay, France) and transferred from the gel onto a polyvinylidene fluoride membrane. The membrane was incubated with the appropriate primary and secondary antibodies described above. The protein of interest was visualized using SuperSignal West Femto maximum sensitivity substrate (Invitrogen).

### Subcellular fractions of the mouse liver

Liver tissue (50 mg) obtained from mice was washed twice with minimal essential medium. Subcellular fractions were prepared using the ProteoExtract subcellular proteome extraction kit (Millipore, Billerica, MA, USA) according to the manufacturer’s protocol. Cytosolic, organelle/membrane, and nuclear proteins were collected into fractions 1 (F1), 2 (F2), and 3 (F3), respectively. The protein concentrations were quantified using the DC protein assay. Each fraction (3 μg protein) was separated using SDS-PAGE, followed by western blotting.

### Nano liquid chromatography (LC)-mass spectrometry (MS)/MS analysis

The immunoprecipitation assay was performed using protein A dynabeads (Thermo Fisher Scientific, Waltham, MA, USA) according to the manufacturer’s protocol. The dynabeads were coupled with IgG2a isotype control or S16 and incubated with the liver lysates obtained from mice at room temperature for 15 min. The immunoprecipitated proteins were separated using 12% SDS-PAGE and visualized using Coomassie Brilliant Blue (CBB) staining. The protein band of interest was cut and processed via in-gel digestion. Briefly, proteins in the gel were reduced and alkylated using dithiothreitol and iodoacetamide, respectively. The proteins were then digested with trypsin and extracted as peptides from each gel. The digested peptides were purified using C18 spin columns (Thermo Fisher Scientific), dried in a SpeedVac concentrator, and dissolved in 5% acetonitrile (ACN) containing 0.5% trifluoroacetic acid. Alternatively, the beads after immunoprecipitation were directly digested with trypsin and analyzed (on-bead digestion).

The tryptic peptides were analyzed using LTQ Orbitrap XL with an ETD mass spectrometer (Thermo Fisher Scientific). The peptides were separated on an L-column ODS at a flow rate of 300 nL/min, with a gradient generated using 0.1% formic acid in water (solvent A) and ACN (solvent B). The gradient for in-gel digestion was as follows: 5–35% B for 30 min, 90% B for 2 min, and 90% B for 8 min. After the LC gradient, data were acquired for 70 and 40 min. Full-scan MS spectra (from *m/z* 300 to 2000) were acquired in the Orbitrap, with a resolution of 30,000 at *m/z* 400 with the lock mass option (m/z at 391.284290 and 445.120030), followed by MS/MS fragmentation in the linear ion trap with a normalized collision energy of 30% against 10 most intense precursor ions with + 2 or more positive charges. Precursor ions for fragmentation were excluded from selection for 10 s. MS/MS data were analyzed using Proteome Discoverer 1.3 (Thermo Fisher Scientific) according to the manufacturer’s instructions and searched against the mouse UniProt database for protein identification.

### Expression and purification of the ACAA2-FLAG recombinant protein

Construction of expression vectors and production of recombinant proteins were performed according to our previous study^42^. The cDNA encoding full-length mouse ACAA2 tagged with a FLAG epitope (ACAA2-FLAG) was cloned into the mammalian expression vector pCAGGS. HEK293 cells were transfected with pCAGGS vector alone or the expression vector encoding ACAA2-FLAG using the TransIT-LT1 reagent (Mirus, Madison, WI, USA) and incubated at 37 °C for 48 h. The cells were lysed using protein extraction buffer at 4 °C for 2 h. The cell lysate was incubated with α-FLAG M2 agarose overnight at 4 °C, followed by elution using 3 × FLAG peptide (Sigma-Aldrich). The concentration of the eluted protein was quantified using a DC protein assay, and the protein was subjected to western blotting. The primer sequences and polymerase chain reaction protocols will be provided upon request.

### Animal treatment

Hepatic injury was induced in mice by administering acetaminophen (APAP) (Sigma-Aldrich). APAP was dissolved in saline (Otsuka Pharmaceutical Factory Inc., Tokushima, Japan) at 42 °C and maintained at 37 °C until use. All mice were fasted for 16 h prior to the experiments. At 0 h, 0.8 mL saline (normal group) or 300 mg of APAP/kg/0.8 mL saline (treatment group) was administered to the peritoneal cavity of mice (see Fig. 6a). The mice were sacrificed to collect blood and livers at 4, 8, 28, and 52 h after APAP treatment.

To analyze the effect of S16 on hepatic injury, APAP-administered mice were divided into three additional groups (five mice per group) after 4 h. Mice in each group were injected intraperitoneally with saline (saline group), 100 μg IgG2a isotype control (IgG2a group), and 100 μg S16 (S16 group) (200 μL/mouse). In the normal group, mice were injected intraperitoneally with 200 μL saline/mouse after 4 h. The mice were sacrificed to collect blood and liver 28 h after APAP treatment.

### Measurements of serum aminotransferase

Serum samples were collected from mice with or without APAP administration. Serum alanine aminotransferase (ALT) levels were measured using colorimetric slides and an automated clinical chemistry analyzer (Fuji Dri-Chem NX 500v; Fujifilm Co. Ltd., Tokyo, Japan).

### ELISA

Serum ACAA2 level was detected using ELISA. Each well of a 96-well plate (Nunc, Roskilde, Denmark) was coated with serum samples (1:10) overnight at 4 °C. These wells were then washed twice with PBS containing 0.05% Tween 20 (PBS-T) and blocked with 1% bovine serum albumin (BSA) for 3 h. After three washes with 0.05% PBS-T, each well was incubated with 1:1,000 dilution of rabbit α-mouse ACAA2 pAb at room temperature for 1 h. The wells were then washed thrice with 0.05% PBS-T and incubated with 1:6,000 dilution of HRP-conjugated goat α-rabbit IgG at room temperature for 1 h. After three washes with 0.05% PBS-T, color was developed by adding TMB + substrate-chromogen (Dako, Carpinteria, CA, USA), and the reaction was terminated using 0.5 M H_2_SO_4_. The plates were then read at 450 nm using a microplate reader (Tecan, Männedorf, Switzerland). The arbitrary cut-off value of ELISA was set at an OD_450_ of 0.1.

Immune complexes formed between S16 and ACAA2 in the serum were detected according to the protocol of Croce et al.^43^ with minor modifications. Rabbit α-mouse ACAA2 pAb (1 μg) was adsorbed in each well overnight at 4 °C. After blocking with 3% BSA at 37 °C for 3 h, serum samples (1:10) were incubated overnight at 4 °C. The wells were washed five times with 0.05% PBS-T and thrice with PBS containing 1% Triton X-100 for 5 min. To remove excess detergent, the wells were washed thrice with 0.05% PBS-T and incubated with 1:6,000 dilution of HRP-conjugated goat α-mouse IgG2a at room temperature for 1 h. After five washes with 0.05% PBS-T, absorbance at 450 nm was measured as described above.

### Histopathology and quantification of necrotic areas in the mouse liver

The livers of five mice per group were harvested and fixed in 20% buffered formalin solution (Muto Chemicals, Tokyo, Japan). The formalin-fixed liver was dehydrated using a series of graded ethanol (concentrations at 70%, 80%, 90%, 95%, and 100%) and embedded in paraffin. The specimens were then stained with hematoxylin and eosin (H&E). The necrotic area of each mouse liver was quantified using hybrid cell count with a BZ-X analyzer (Keyence Corporation, Osaka, Japan) for two different images per section.

### Statistical analysis

A two-tailed unpaired Student’s *t*-test was conducted to analyze the statistical significance of the difference between groups when necessary. Differences were considered significant at *p* < 0.05.

### Ethics statement

Animal experiments were performed in accordance with the guidelines for the Proper Conduct of Animal Experiments established by the Science Council of Japan and were approved by the Committee on the Ethics of Animal Experiments (CEAE) at the Iwate Medical University. All animal experiments were performed in accordance with the guidelines set by the Iwate Medical University CEAE (permit numbers 29-029).

## Supplementary Information

Supplementary Information.
