# Supplementary material for: A mouse monoclonal antibody against influenza C virus attenuates acetaminophen-induced liver injury in mice
Source: Sci Rep. 2021 Jun 3;11:11816. doi: 10.1038/s41598-021-91251-x (PMC8175586; doi:10.1038/s41598-021-91251-x)
Supplement: Supplementary file 1 — Supplementary Information. [file 41598_2021_91251_MOESM1_ESM.pptx]

## Slide 1
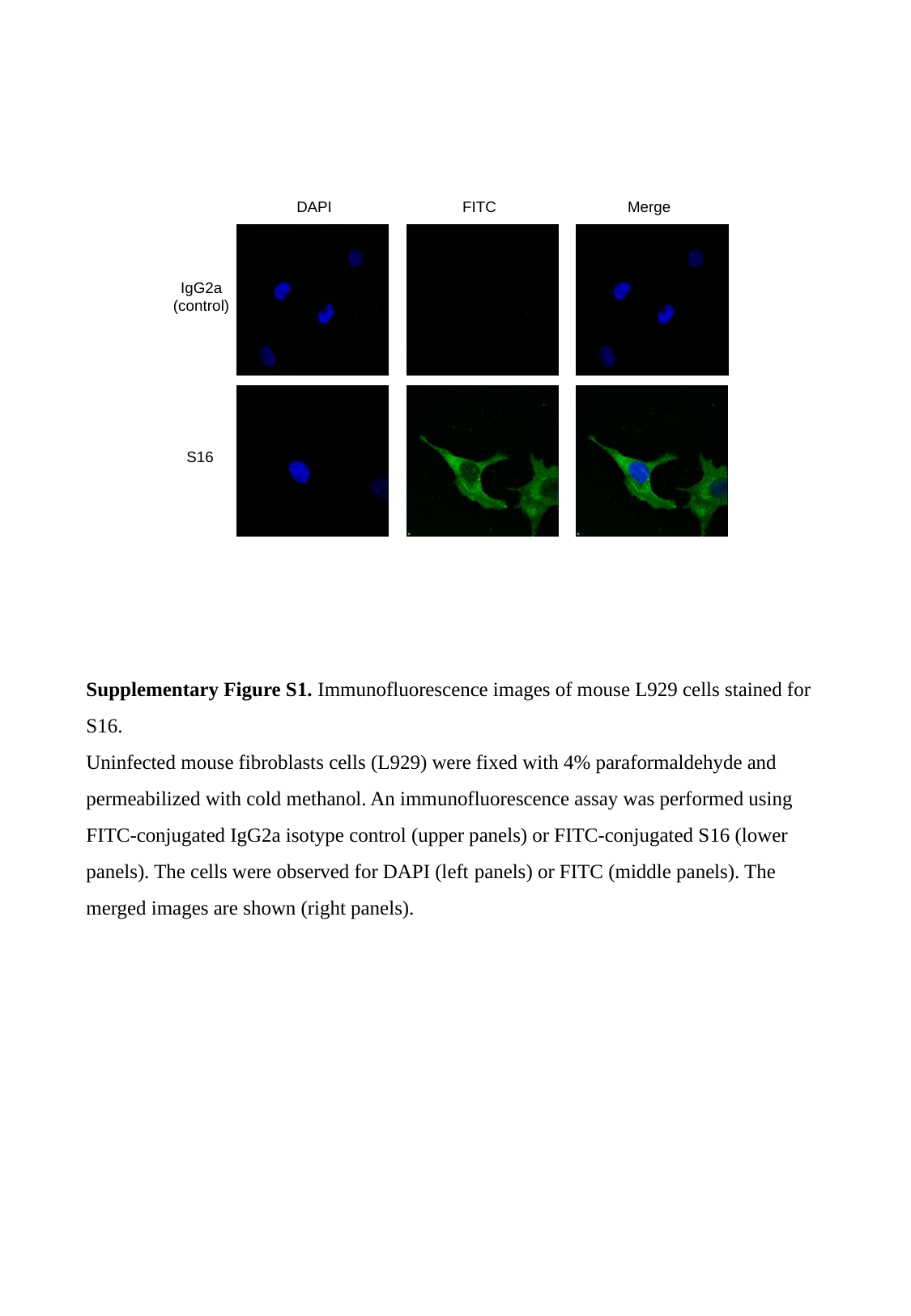

DAPI
FITC
Merge
IgG2a (control)
S16
Supplementary Figure S1. Immunofluorescence images of mouse L929 cells stained for S16.
Uninfected mouse fibroblasts cells (L929) were fixed with 4% paraformaldehyde and permeabilized with cold methanol. An immunofluorescence assay was performed using FITC-conjugated IgG2a isotype control (upper panels) or FITC-conjugated S16 (lower panels). The cells were observed for DAPI (left panels) or FITC (middle panels). The merged images are shown (right panels).

## Slide 2
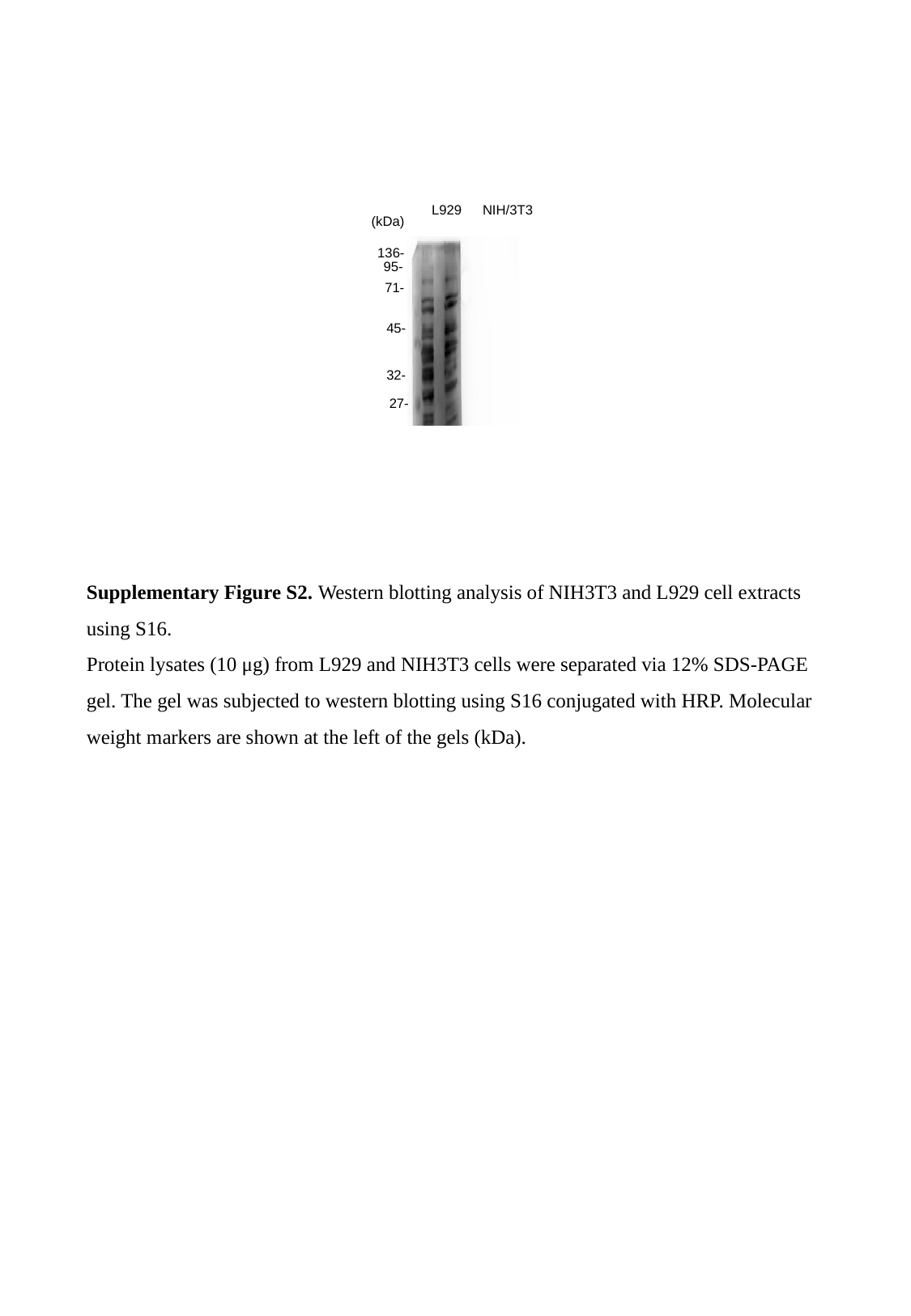

L929
NIH/3T3
(kDa)
136-
95-
71-
45-
32-
27-
Supplementary Figure S2. Western blotting analysis of NIH3T3 and L929 cell extracts using S16.
Protein lysates (10 μg) from L929 and NIH3T3 cells were separated via 12% SDS-PAGE gel. The gel was subjected to western blotting using S16 conjugated with HRP. Molecular weight markers are shown at the left of the gels (kDa).

## Slide 3
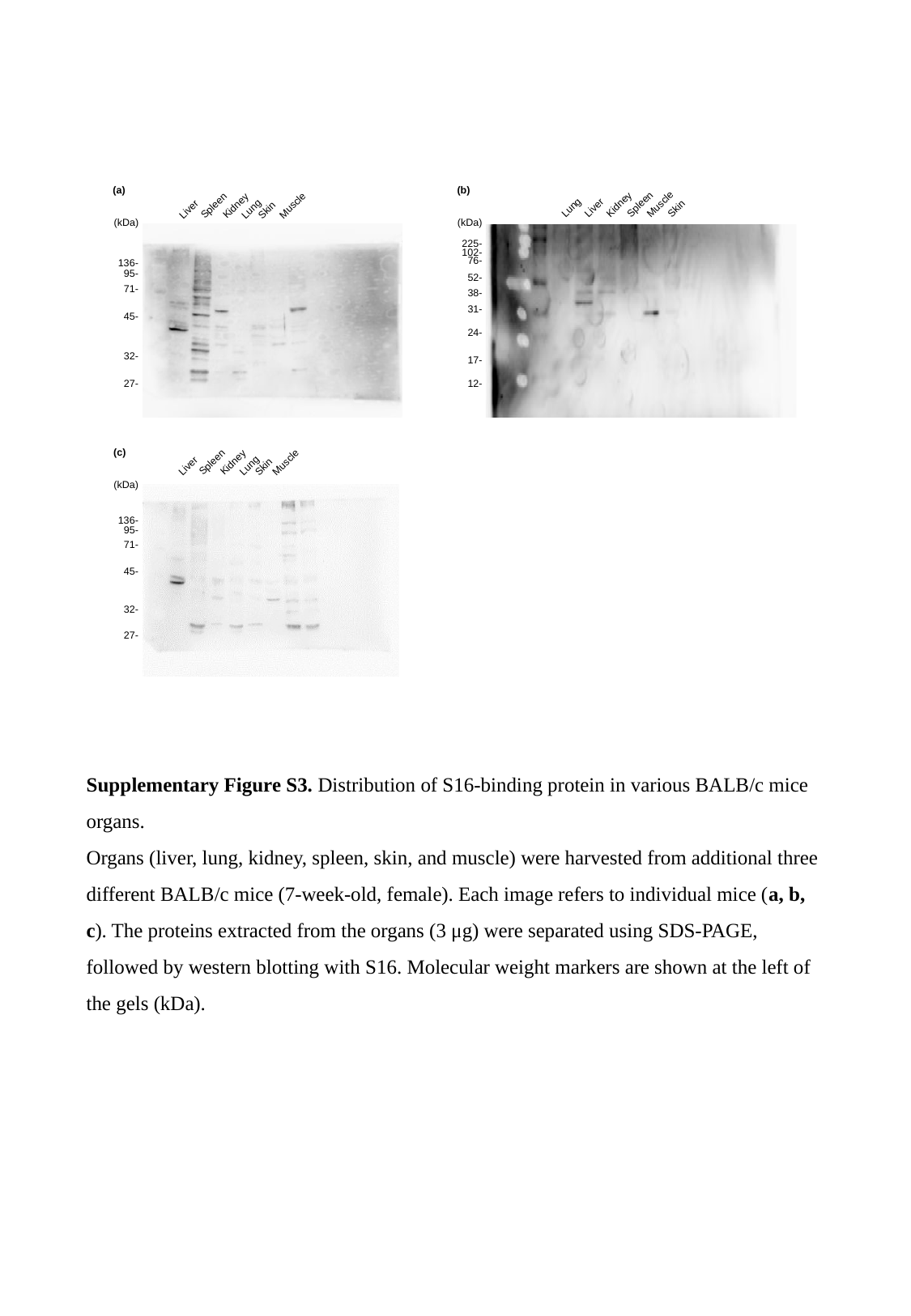

(a)
Muscle
Spleen
Kidney
Lung
Liver
Skin
(kDa)
136-
95-
71-
45-
32-
27-
(b)
Muscle
Spleen
Kidney
Lung
Liver
Skin
(kDa)
225-
102-
76-
52-
38-
31-
24-
17-
12-
(c)
Muscle
Spleen
Kidney
Lung
Liver
Skin
(kDa)
136-
95-
71-
45-
32-
27-
Supplementary Figure S3. Distribution of S16-binding protein in various BALB/c mice organs.
Organs (liver, lung, kidney, spleen, skin, and muscle) were harvested from additional three different BALB/c mice (7-week-old, female). Each image refers to individual mice (a, b, c). The proteins extracted from the organs (3 μg) were separated using SDS-PAGE, followed by western blotting with S16. Molecular weight markers are shown at the left of the gels (kDa).

## Slide 4
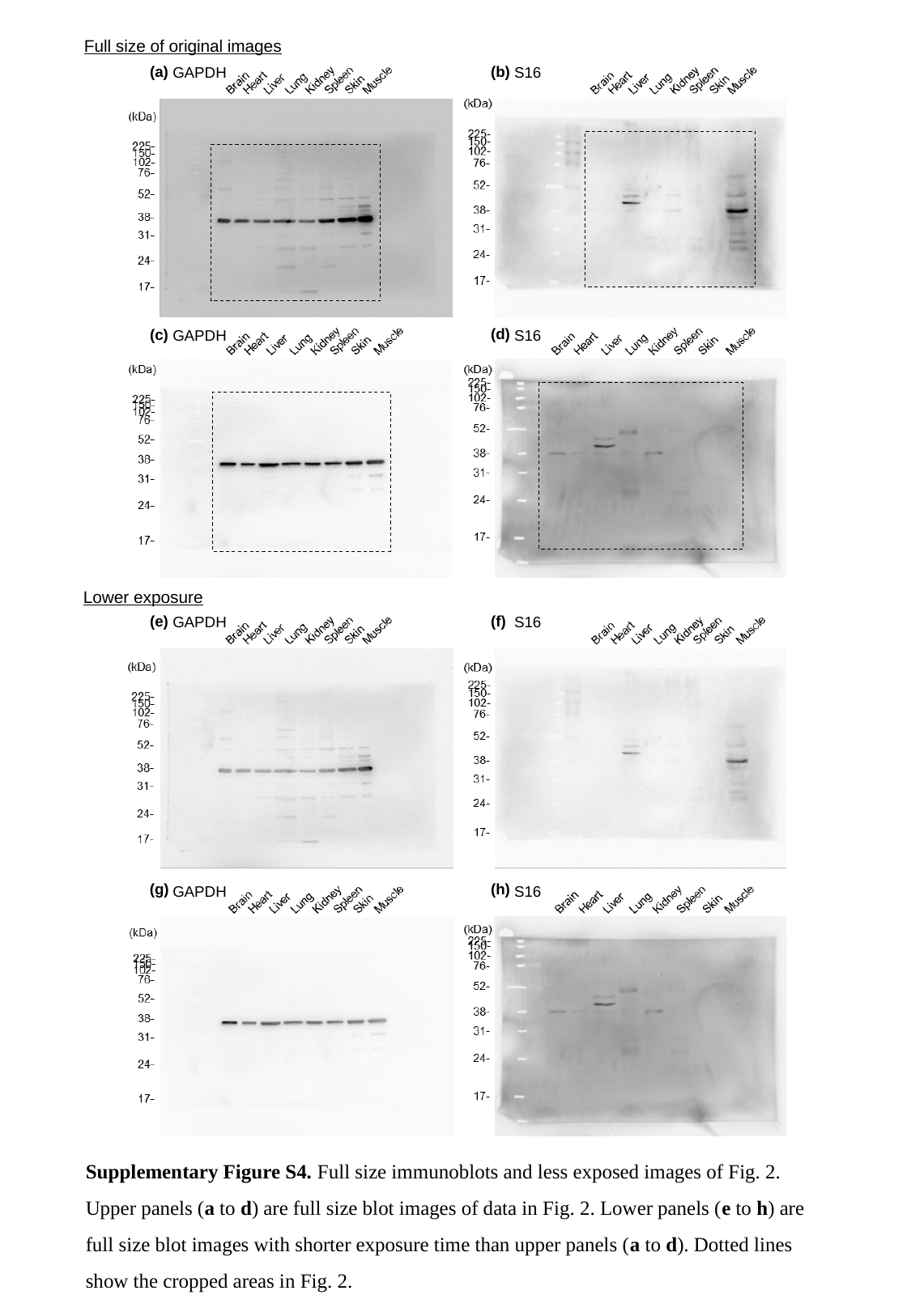

Full size of original images
(a)
(b)
GAPDH
S16
(d)
(c)
GAPDH
S16
Lower exposure
(e)
(f)
GAPDH
S16
(g)
(h)
GAPDH
S16
Supplementary Figure S4. Full size immunoblots and less exposed images of Fig. 2.
Upper panels (a to d) are full size blot images of data in Fig. 2. Lower panels (e to h) are full size blot images with shorter exposure time than upper panels (a to d). Dotted lines show the cropped areas in Fig. 2.

## Slide 5
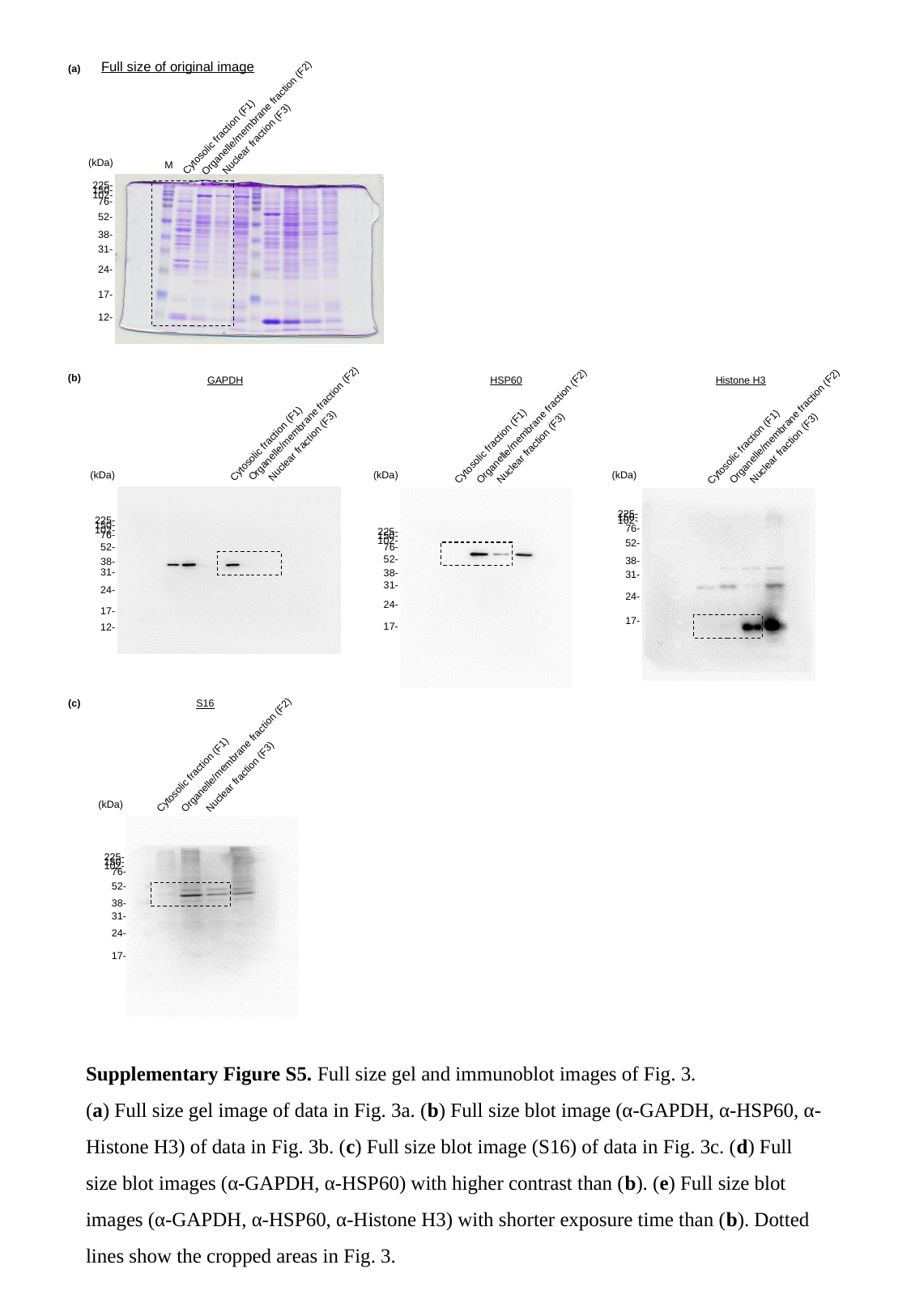

Full size of original image
(a)
Organelle/membrane fraction (F2)
Cytosolic fraction (F1)
Nuclear fraction (F3)
(kDa)
M
225-
150-
102-
76-
52-
38-
31-
24-
17-
12-
(b)
GAPDH
HSP60
Histone H3
Organelle/membrane fraction (F2)
Organelle/membrane fraction (F2)
Organelle/membrane fraction (F2)
Cytosolic fraction (F1)
Nuclear fraction (F3)
Cytosolic fraction (F1)
Cytosolic fraction (F1)
Nuclear fraction (F3)
Nuclear fraction (F3)
(kDa)
(kDa)
(kDa)
225-
150-
102-
225-
150-
76-
102-
225-
76-
150-
102-
52-
76-
52-
52-
38-
38-
31-
38-
31-
31-
24-
24-
24-
17-
17-
17-
12-
(c)
S16
Organelle/membrane fraction (F2)
Cytosolic fraction (F1)
Nuclear fraction (F3)
(kDa)
225-
150-
102-
76-
52-
38-
31-
24-
17-
Supplementary Figure S5. Full size gel and immunoblot images of Fig. 3.
(a) Full size gel image of data in Fig. 3a. (b) Full size blot image (α-GAPDH, α-HSP60, α-Histone H3) of data in Fig. 3b. (c) Full size blot image (S16) of data in Fig. 3c. (d) Full size blot images (α-GAPDH, α-HSP60) with higher contrast than (b). (e) Full size blot images (α-GAPDH, α-HSP60, α-Histone H3) with shorter exposure time than (b). Dotted lines show the cropped areas in Fig. 3.

## Slide 6
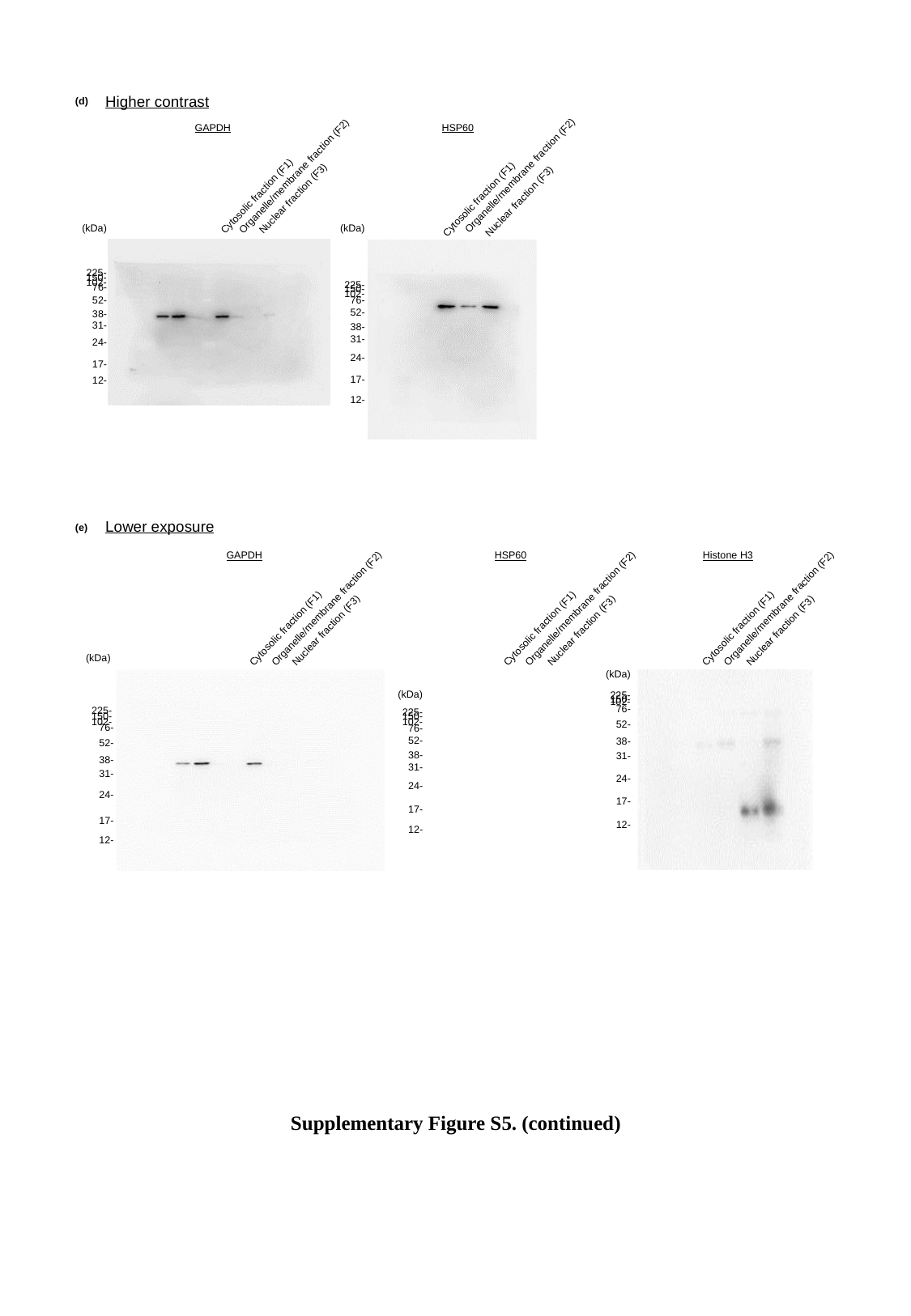

Higher contrast
(d)
GAPDH
HSP60
Organelle/membrane fraction (F2)
Organelle/membrane fraction (F2)
Cytosolic fraction (F1)
Nuclear fraction (F3)
Cytosolic fraction (F1)
Nuclear fraction (F3)
(kDa)
(kDa)
225-
150-
102-
225-
76-
150-
102-
52-
76-
52-
38-
31-
38-
31-
24-
24-
17-
17-
12-
12-
Lower exposure
(e)
GAPDH
HSP60
Histone H3
Organelle/membrane fraction (F2)
Organelle/membrane fraction (F2)
Organelle/membrane fraction (F2)
Cytosolic fraction (F1)
Cytosolic fraction (F1)
Cytosolic fraction (F1)
Nuclear fraction (F3)
Nuclear fraction (F3)
Nuclear fraction (F3)
(kDa)
(kDa)
(kDa)
225-
150-
102-
76-
225-
225-
150-
150-
102-
102-
52-
76-
76-
52-
38-
52-
38-
31-
38-
31-
31-
24-
24-
24-
17-
17-
17-
12-
12-
12-
Supplementary Figure S5. (continued)

## Slide 7
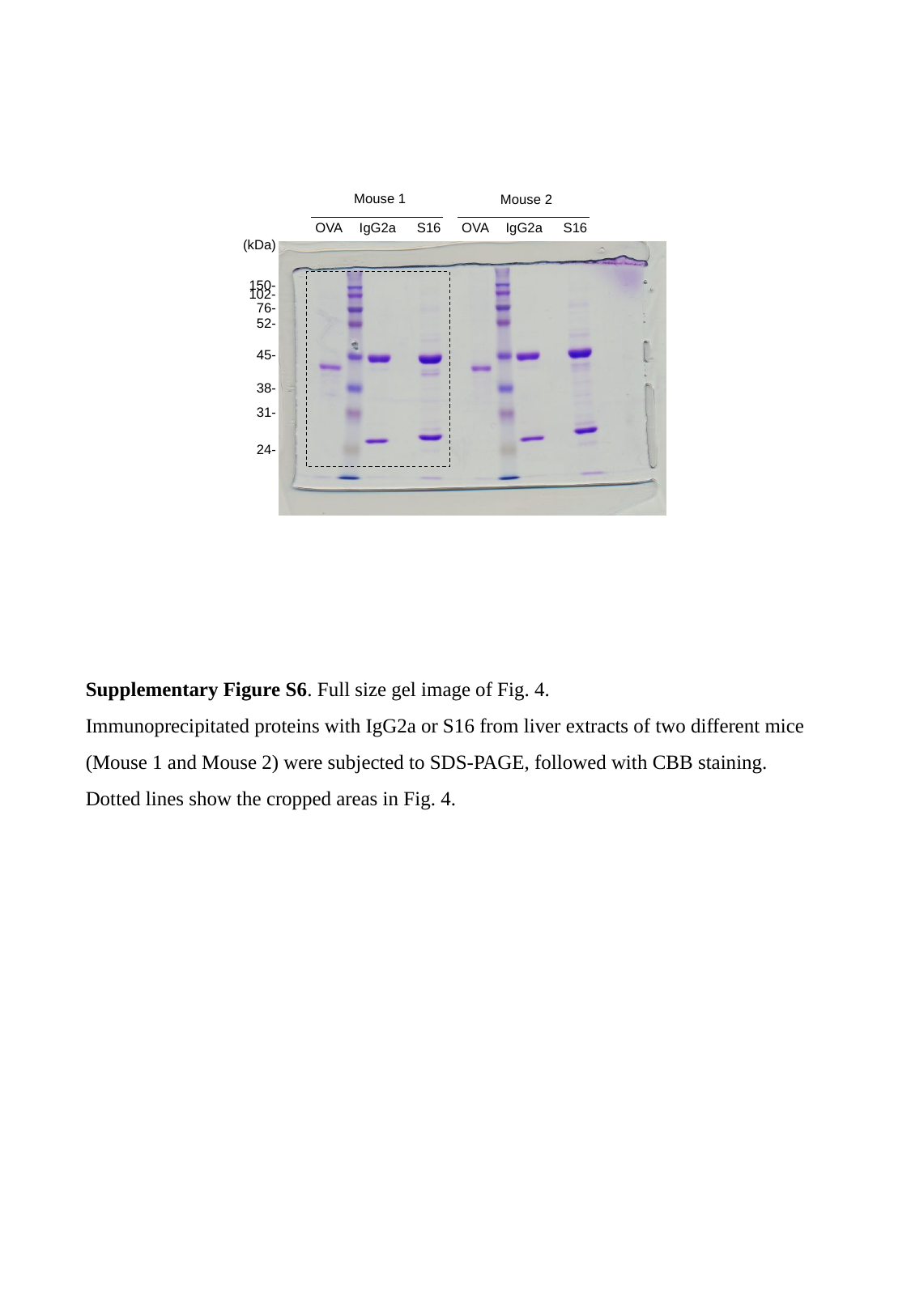

Mouse 1
Mouse 2
OVA
IgG2a
S16
OVA
IgG2a
S16
(kDa)
150-
102-
76-
52-
45-
38-
31-
24-
Supplementary Figure S6. Full size gel image of Fig. 4.
Immunoprecipitated proteins with IgG2a or S16 from liver extracts of two different mice (Mouse 1 and Mouse 2) were subjected to SDS-PAGE, followed with CBB staining. Dotted lines show the cropped areas in Fig. 4.

## Slide 8
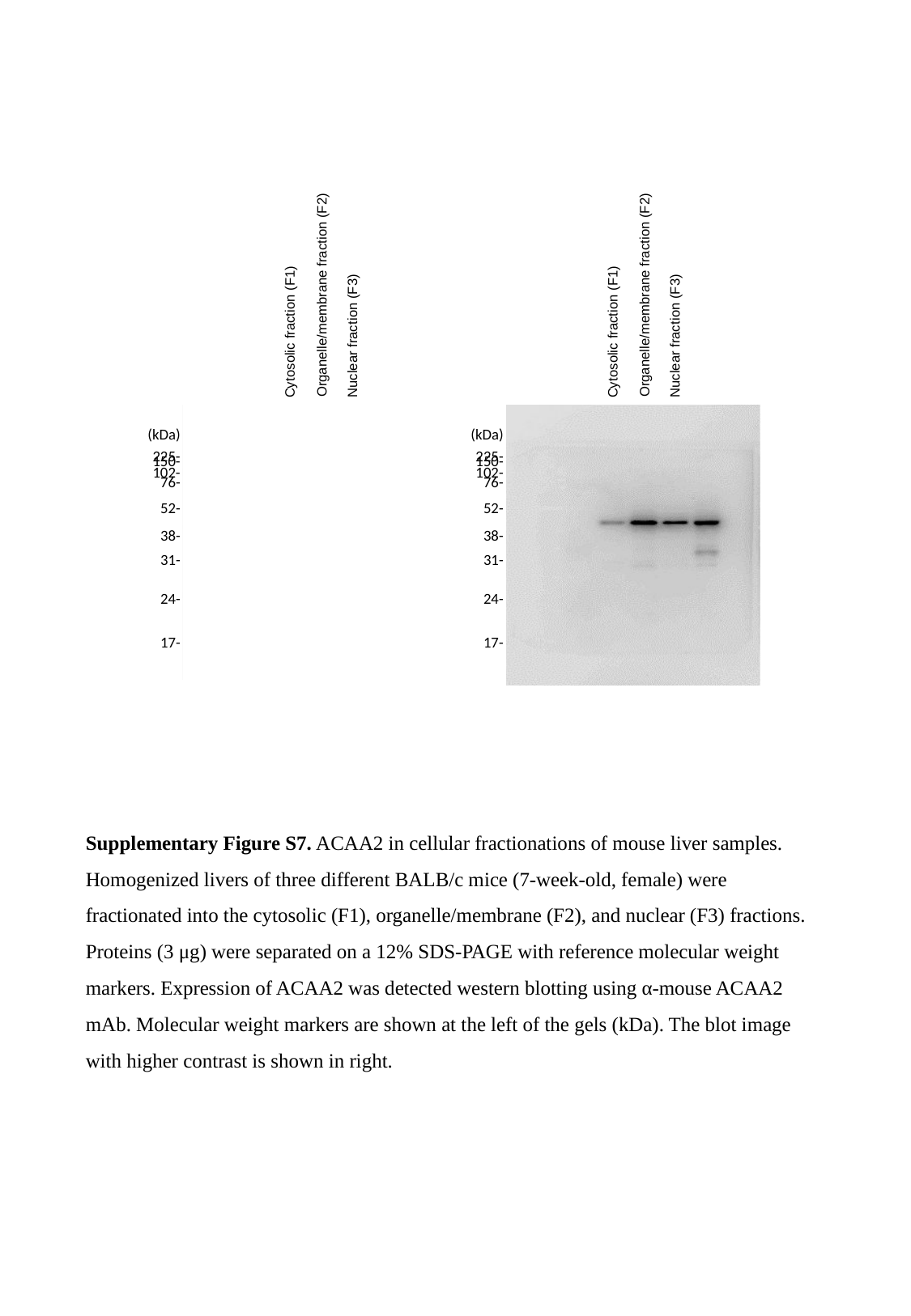

Organelle/membrane fraction (F2)
Cytosolic fraction (F1)
Nuclear fraction (F3)
(kDa)
225-
150-
102-
76-
52-
38-
31-
24-
17-
Organelle/membrane fraction (F2)
Cytosolic fraction (F1)
Nuclear fraction (F3)
(kDa)
225-
150-
102-
76-
52-
38-
31-
24-
17-
Supplementary Figure S7. ACAA2 in cellular fractionations of mouse liver samples.
Homogenized livers of three different BALB/c mice (7-week-old, female) were fractionated into the cytosolic (F1), organelle/membrane (F2), and nuclear (F3) fractions. Proteins (3 μg) were separated on a 12% SDS-PAGE with reference molecular weight markers. Expression of ACAA2 was detected western blotting using α-mouse ACAA2 mAb. Molecular weight markers are shown at the left of the gels (kDa). The blot image with higher contrast is shown in right.

## Slide 9
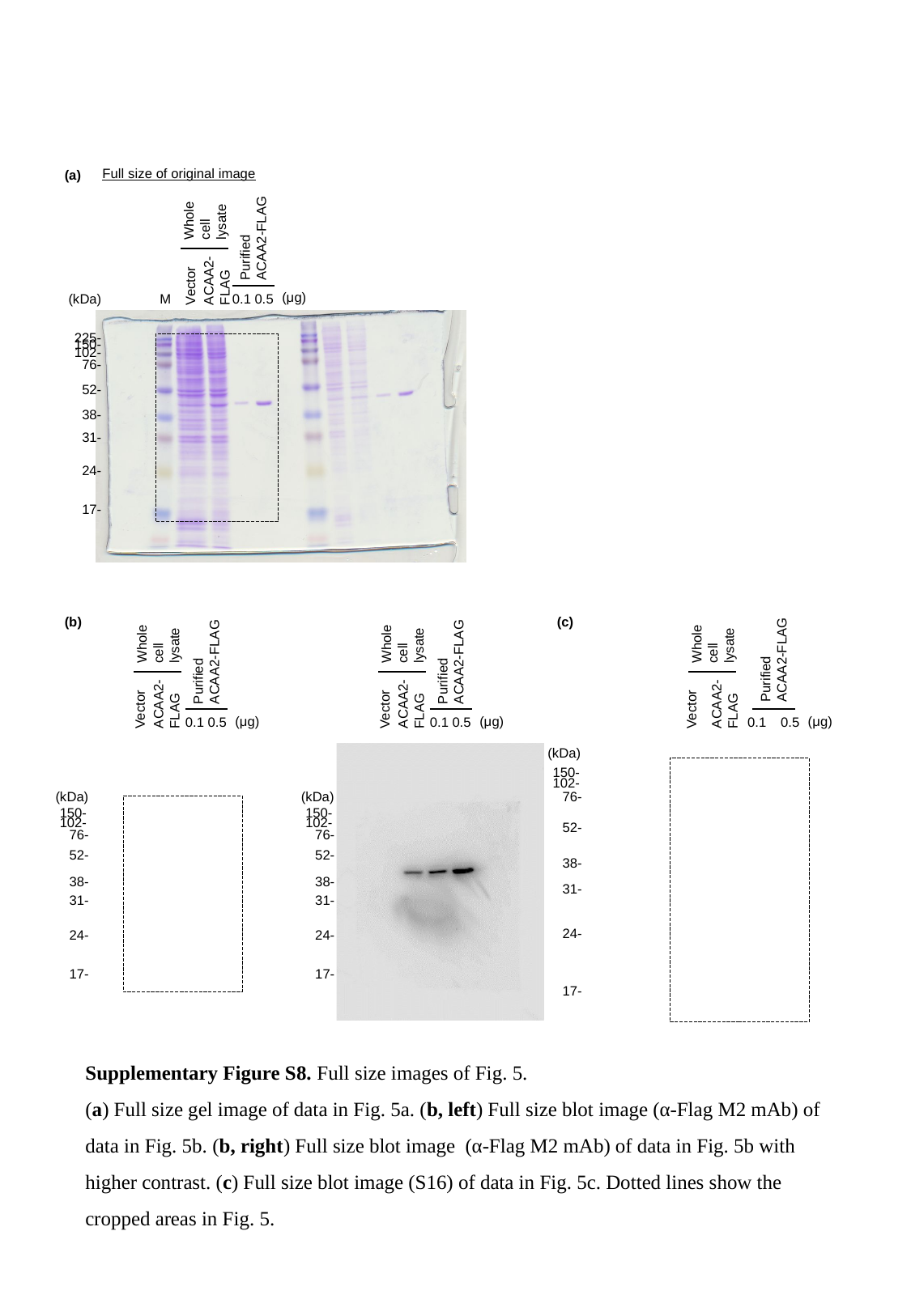

Full size of original image
(a)
Whole cell lysate
Purified
ACAA2-FLAG
ACAA2-
FLAG
Vector
(μg)
M
0.1
0.5
(kDa)
225-
150-
102-
76-
52-
38-
31-
24-
17-
(b)
Whole cell lysate
Purified
ACAA2-FLAG
ACAA2-
FLAG
Vector
(μg)
0.1
0.5
Whole cell lysate
Purified
ACAA2-FLAG
ACAA2-
FLAG
Vector
(μg)
0.1
0.5
(kDa)
(kDa)
150-
150-
102-
102-
76-
76-
52-
52-
38-
38-
31-
31-
24-
24-
17-
17-
(c)
Whole cell lysate
Purified
ACAA2-FLAG
ACAA2-
FLAG
Vector
(μg)
0.1
0.5
(kDa)
150-
102-
76-
52-
38-
31-
24-
17-
Supplementary Figure S8. Full size images of Fig. 5.
(a) Full size gel image of data in Fig. 5a. (b, left) Full size blot image (α-Flag M2 mAb) of data in Fig. 5b. (b, right) Full size blot image (α-Flag M2 mAb) of data in Fig. 5b with higher contrast. (c) Full size blot image (S16) of data in Fig. 5c. Dotted lines show the cropped areas in Fig. 5.

## Slide 10
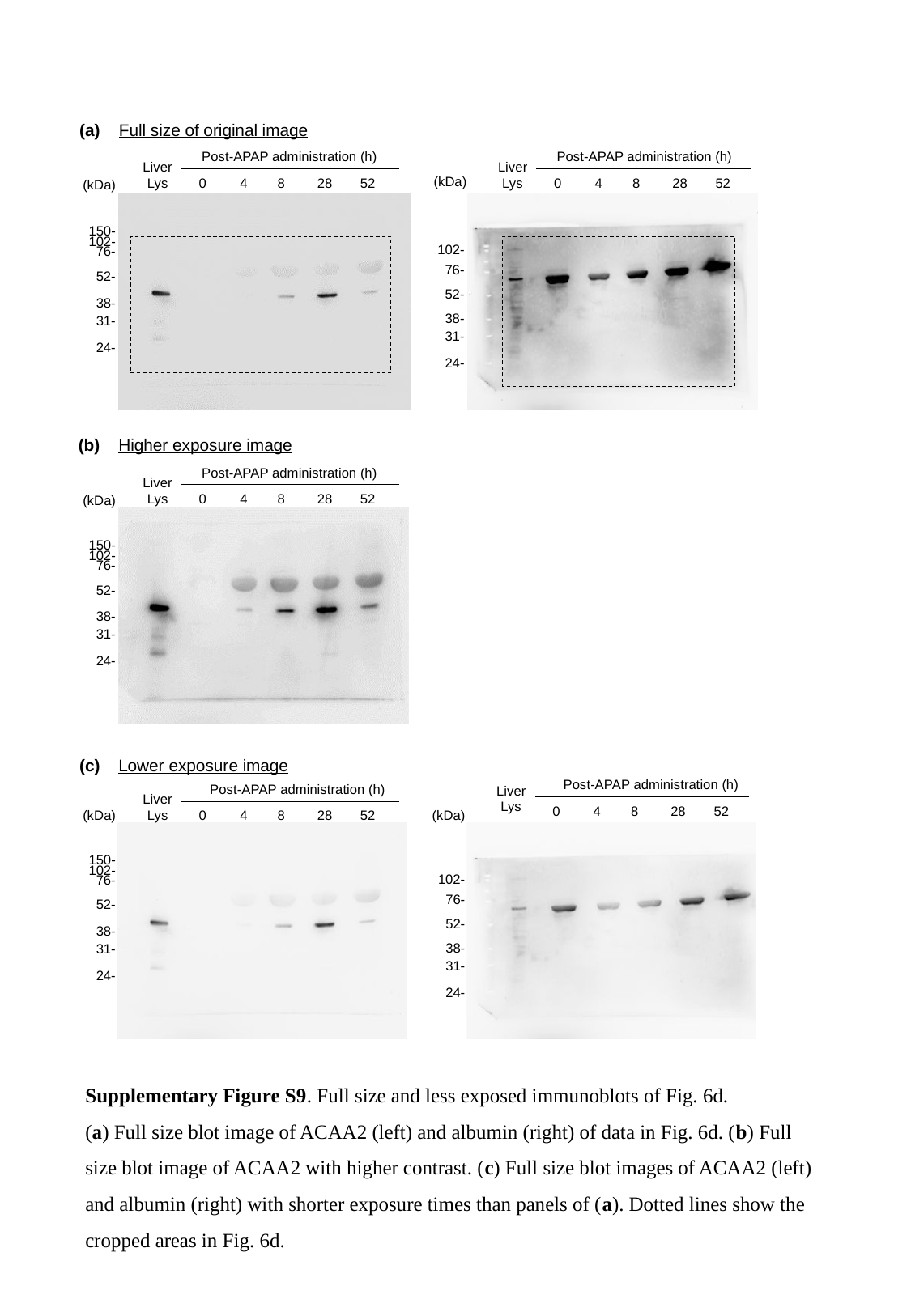

Full size of original image
(a)
Post-APAP administration (h)
Post-APAP administration (h)
Liver
Lys
Liver
Lys
(kDa)
0
4
8
28
52
0
4
8
28
52
(kDa)
150-
102-
102-
76-
76-
52-
52-
38-
38-
31-
31-
24-
24-
Higher exposure image
(b)
Post-APAP administration (h)
Liver
Lys
0
4
8
28
52
(kDa)
150-
102-
76-
52-
38-
31-
24-
Lower exposure image
(c)
Post-APAP administration (h)
Post-APAP administration (h)
Liver
Lys
Liver
Lys
0
4
8
28
52
(kDa)
(kDa)
0
4
8
28
52
150-
102-
102-
76-
76-
52-
52-
38-
38-
31-
31-
24-
24-
Supplementary Figure S9. Full size and less exposed immunoblots of Fig. 6d.
(a) Full size blot image of ACAA2 (left) and albumin (right) of data in Fig. 6d. (b) Full size blot image of ACAA2 with higher contrast. (c) Full size blot images of ACAA2 (left) and albumin (right) with shorter exposure times than panels of (a). Dotted lines show the cropped areas in Fig. 6d.

## Slide 11
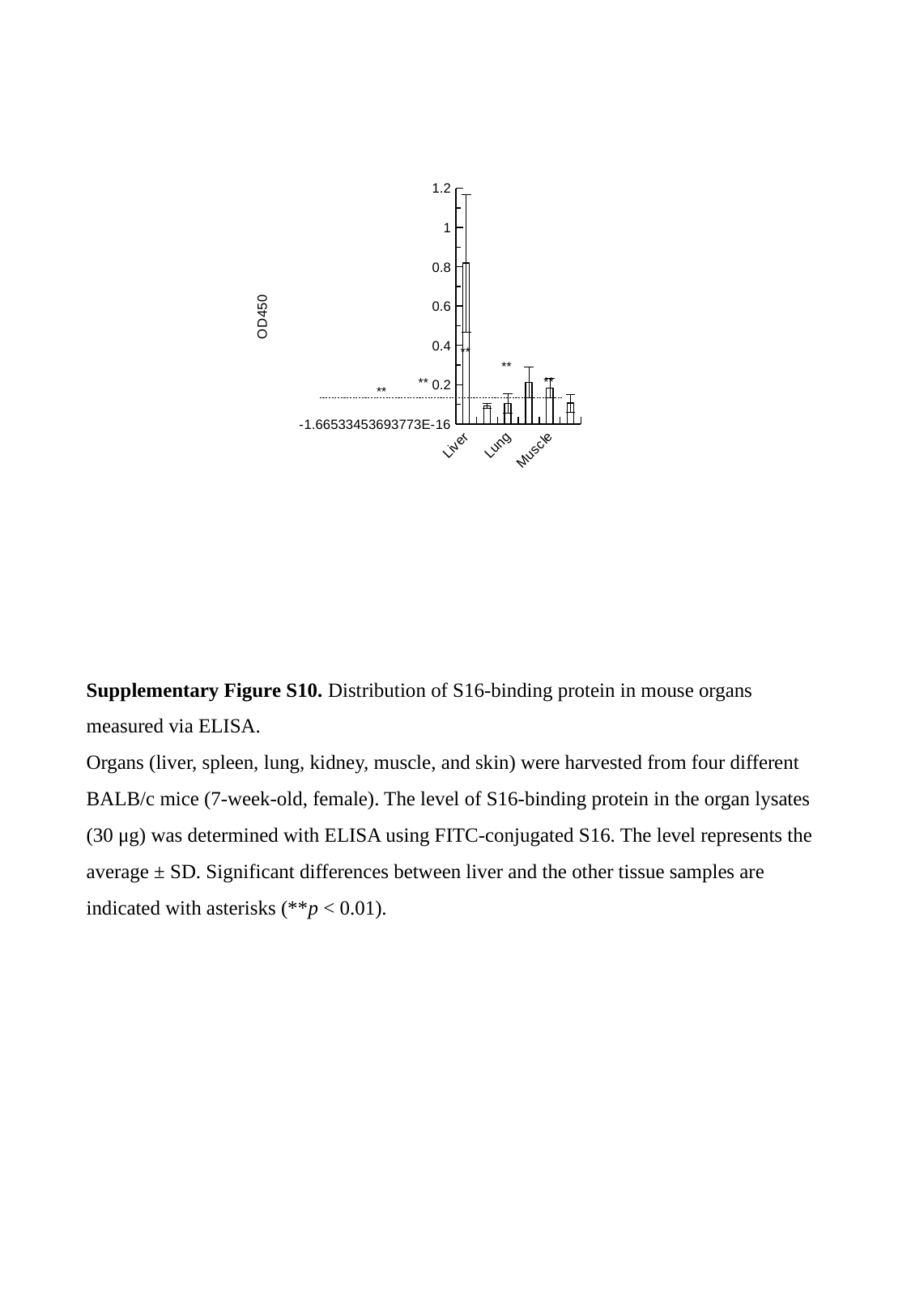

### Chart
| Category | ave |
|---|---|
| Liver | 0.8182499999999999 |
| Spleen | 0.09266666666666667 |
| Lung | 0.10500000000000001 |
| Kidney | 0.21200000000000002 |
| Muscle | 0.1825 |
| Skin | 0.10600000000000001 |**
**
**
**
**
Supplementary Figure S10. Distribution of S16-binding protein in mouse organs measured via ELISA.
Organs (liver, spleen, lung, kidney, muscle, and skin) were harvested from four different BALB/c mice (7-week-old, female). The level of S16-binding protein in the organ lysates (30 μg) was determined with ELISA using FITC-conjugated S16. The level represents the average ± SD. Significant differences between liver and the other tissue samples are indicated with asterisks (**p < 0.01).

## Slide 12
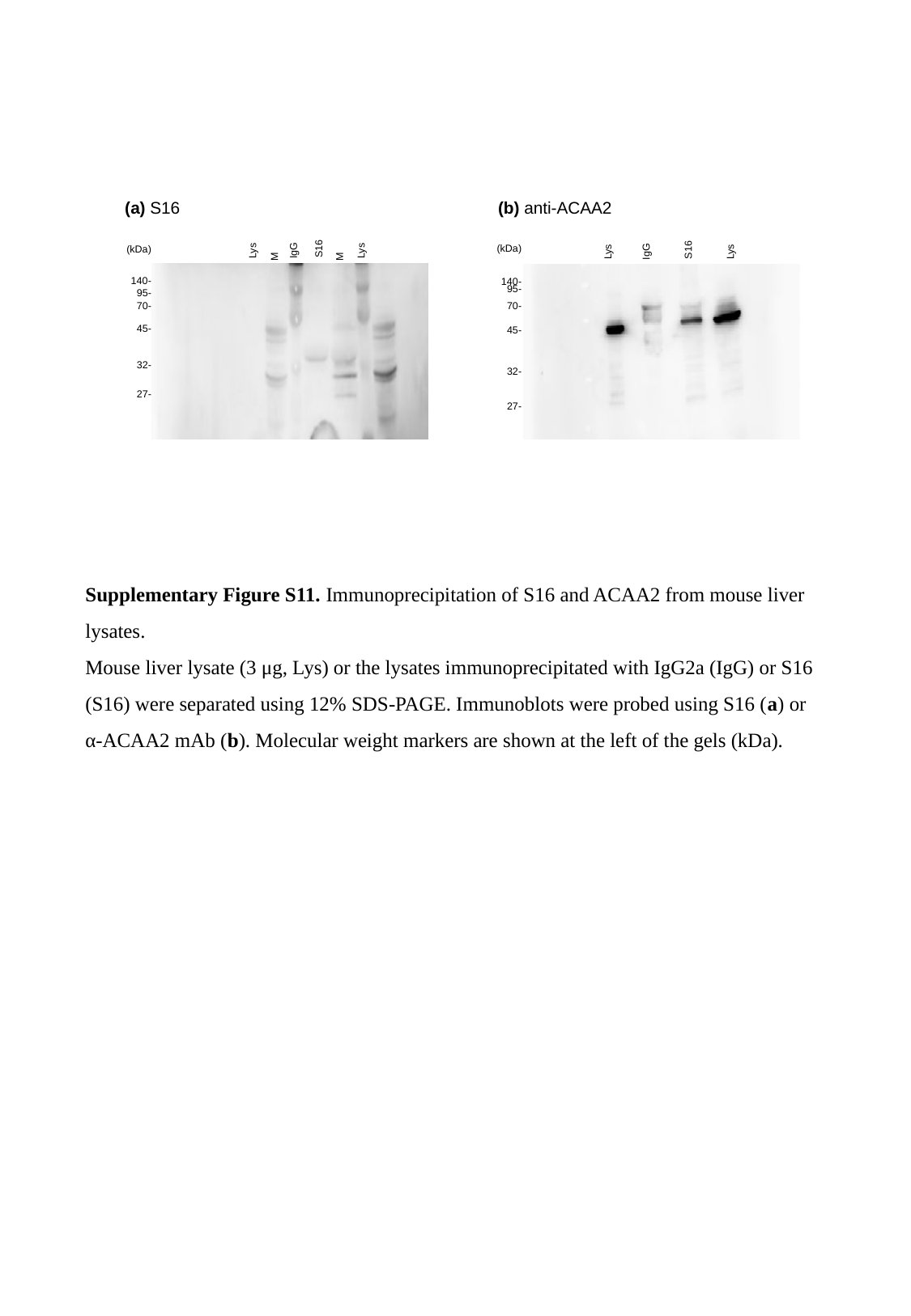

(a) S16
S16
IgG
Lys
Lys
M
M
(kDa)
140-
95-
70-
45-
32-
27-
(b) anti-ACAA2
S16
IgG
Lys
Lys
(kDa)
140-
95-
70-
45-
32-
27-
Supplementary Figure S11. Immunoprecipitation of S16 and ACAA2 from mouse liver lysates.
Mouse liver lysate (3 μg, Lys) or the lysates immunoprecipitated with IgG2a (IgG) or S16 (S16) were separated using 12% SDS-PAGE. Immunoblots were probed using S16 (a) or α-ACAA2 mAb (b). Molecular weight markers are shown at the left of the gels (kDa).

## Slide 13
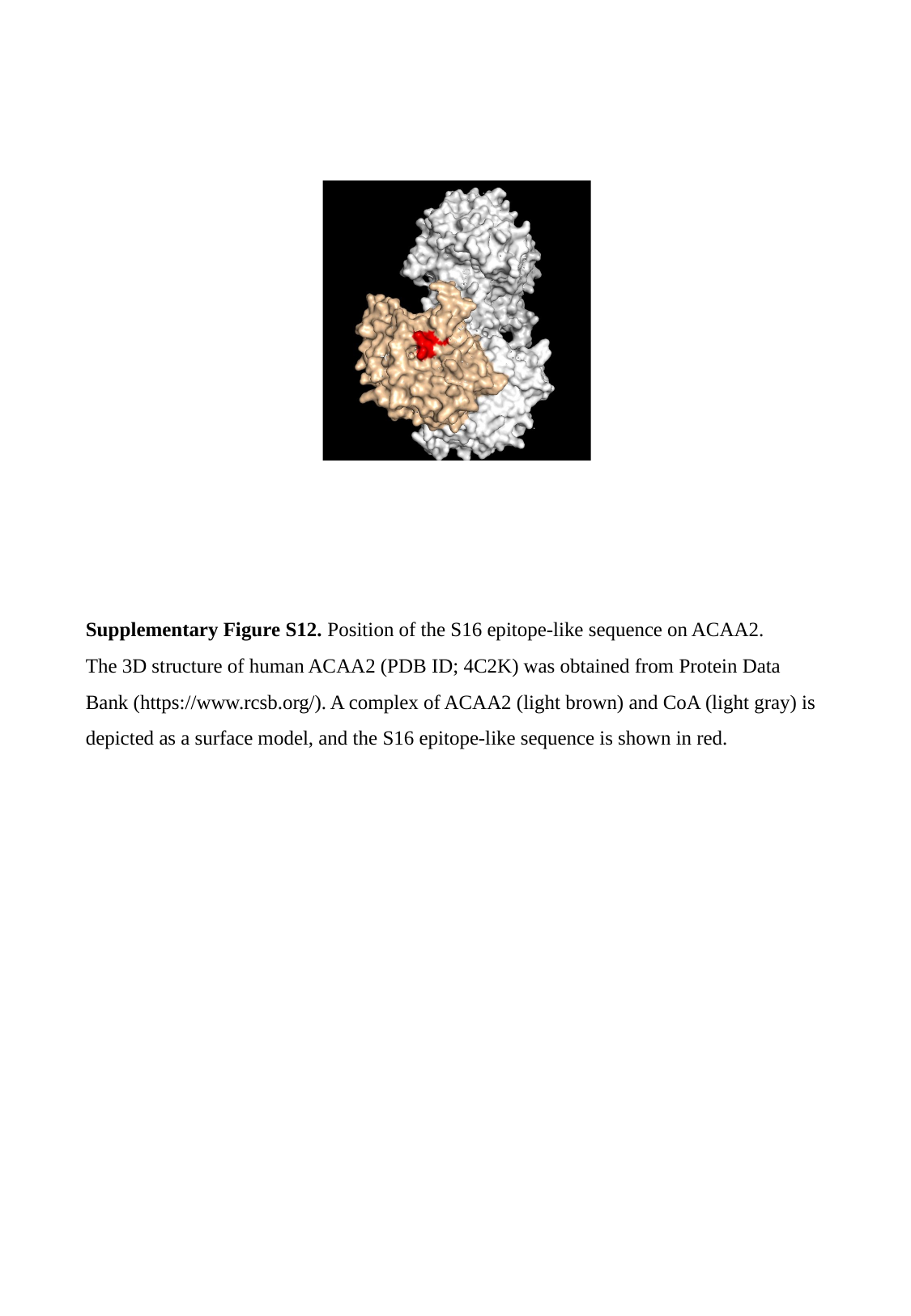

Supplementary Figure S12. Position of the S16 epitope-like sequence on ACAA2.
The 3D structure of human ACAA2 (PDB ID; 4C2K) was obtained from Protein Data Bank (https://www.rcsb.org/). A complex of ACAA2 (light brown) and CoA (light gray) is depicted as a surface model, and the S16 epitope-like sequence is shown in red.

## Slide 14
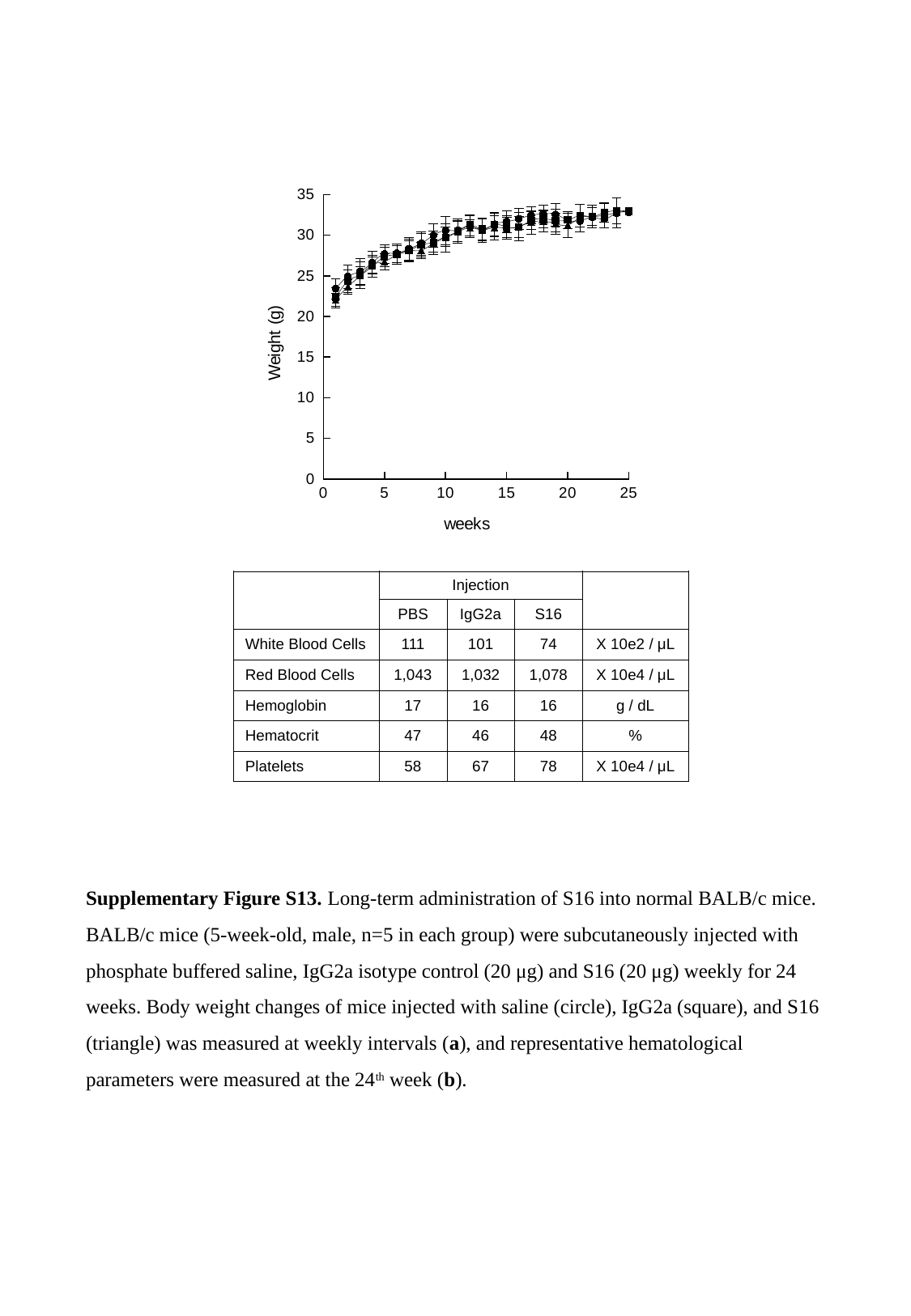

### Chart
| Category | | | 精製S16 |
|---|---|---|---|| | Injection | | | |
| --- | --- | --- | --- | --- |
| | PBS | IgG2a | S16 | |
| White Blood Cells | 111 | 101 | 74 | X 10e2 / μL |
| Red Blood Cells | 1,043 | 1,032 | 1,078 | X 10e4 / μL |
| Hemoglobin | 17 | 16 | 16 | g / dL |
| Hematocrit | 47 | 46 | 48 | % |
| Platelets | 58 | 67 | 78 | X 10e4 / μL |
Supplementary Figure S13. Long-term administration of S16 into normal BALB/c mice.
BALB/c mice (5-week-old, male, n=5 in each group) were subcutaneously injected with phosphate buffered saline, IgG2a isotype control (20 μg) and S16 (20 μg) weekly for 24 weeks. Body weight changes of mice injected with saline (circle), IgG2a (square), and S16 (triangle) was measured at weekly intervals (a), and representative hematological parameters were measured at the 24th week (b).

## Slide 15
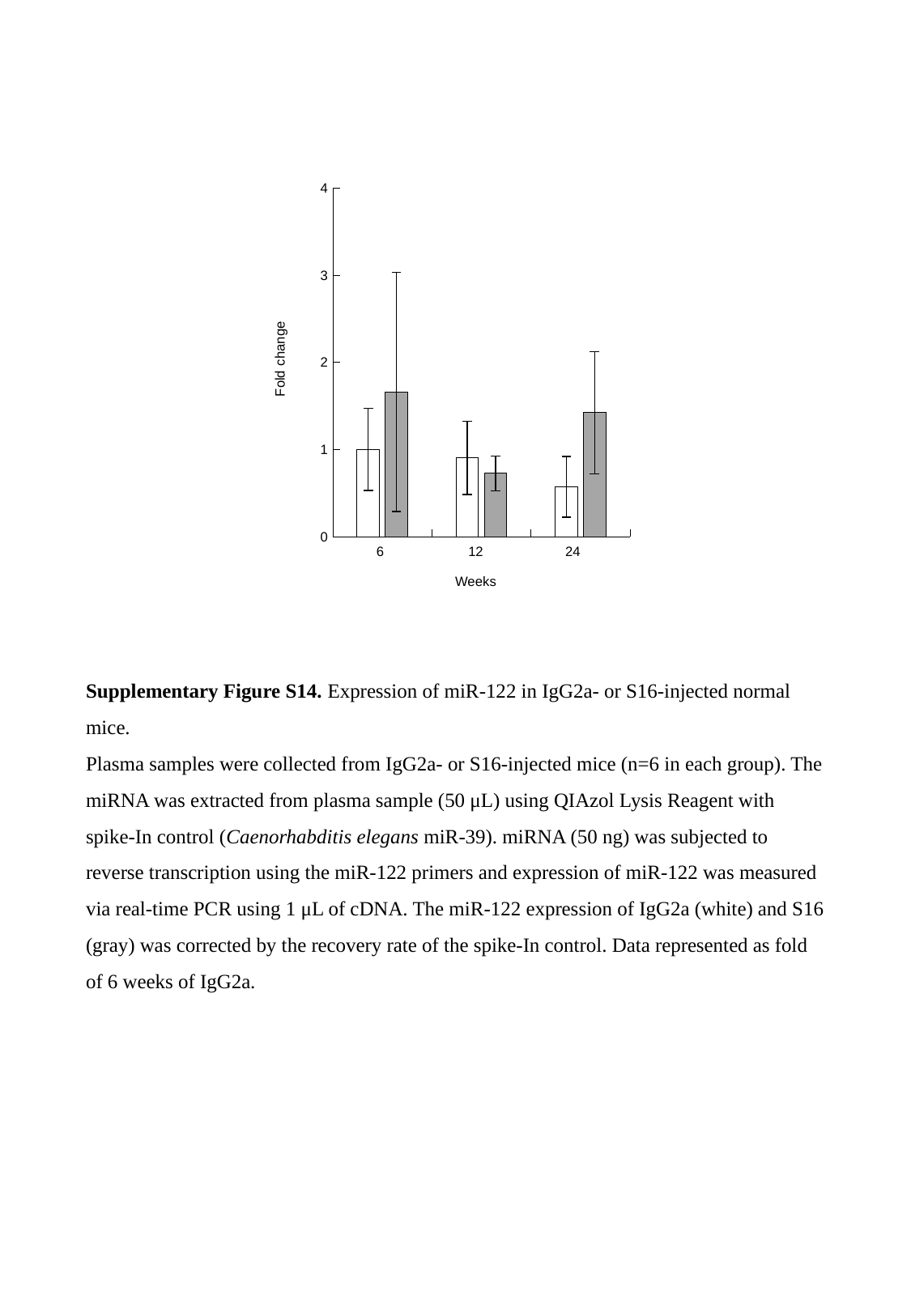

### Chart
| Category | IgG2a | 精製S16 |
|---|---|---|6
12
24
Weeks
Supplementary Figure S14. Expression of miR-122 in IgG2a- or S16-injected normal mice.
Plasma samples were collected from IgG2a- or S16-injected mice (n=6 in each group). The miRNA was extracted from plasma sample (50 μL) using QIAzol Lysis Reagent with spike-In control (Caenorhabditis elegans miR-39). miRNA (50 ng) was subjected to reverse transcription using the miR-122 primers and expression of miR-122 was measured via real-time PCR using 1 μL of cDNA. The miR-122 expression of IgG2a (white) and S16 (gray) was corrected by the recovery rate of the spike-In control. Data represented as fold of 6 weeks of IgG2a.

## Slide 16
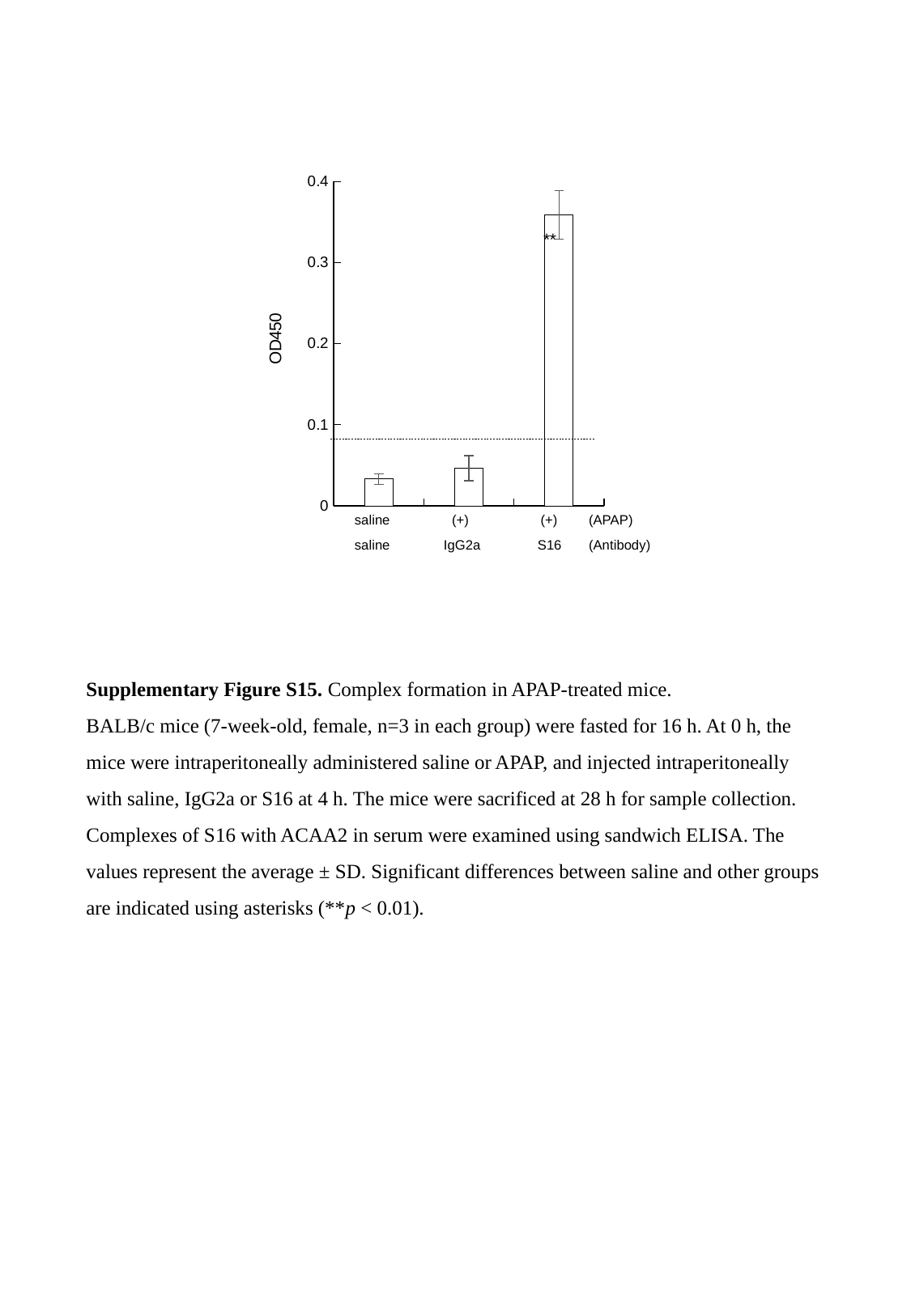

### Chart
| Category | ave |
|---|---|
| 生食 | 0.03300000000000001 |
| IgG2a | 0.04650000000000001 |
| S16 | 0.35875 |**
saline
(+)
(+)
(APAP)
saline
IgG2a
S16
(Antibody)
Supplementary Figure S15. Complex formation in APAP-treated mice.
BALB/c mice (7-week-old, female, n=3 in each group) were fasted for 16 h. At 0 h, the mice were intraperitoneally administered saline or APAP, and injected intraperitoneally with saline, IgG2a or S16 at 4 h. The mice were sacrificed at 28 h for sample collection. Complexes of S16 with ACAA2 in serum were examined using sandwich ELISA. The values represent the average ± SD. Significant differences between saline and other groups are indicated using asterisks (**p < 0.01).

## Slide 17
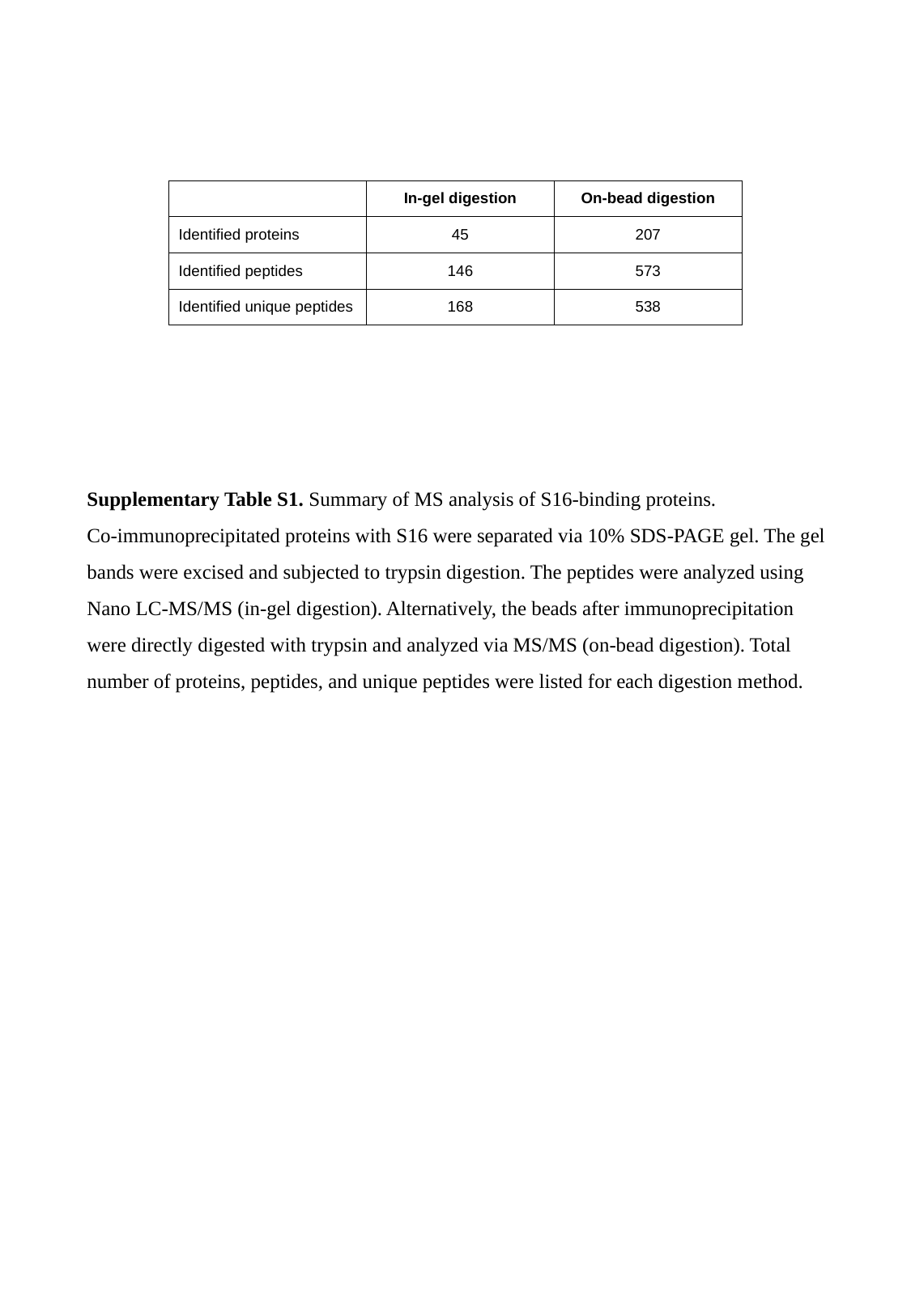

| | In-gel digestion | On-bead digestion |
| --- | --- | --- |
| Identified proteins | 45 | 207 |
| Identified peptides | 146 | 573 |
| Identified unique peptides | 168 | 538 |
Supplementary Table S1. Summary of MS analysis of S16-binding proteins.
Co-immunoprecipitated proteins with S16 were separated via 10% SDS-PAGE gel. The gel bands were excised and subjected to trypsin digestion. The peptides were analyzed using Nano LC-MS/MS (in-gel digestion). Alternatively, the beads after immunoprecipitation were directly digested with trypsin and analyzed via MS/MS (on-bead digestion). Total number of proteins, peptides, and unique peptides were listed for each digestion method.

## Slide 18
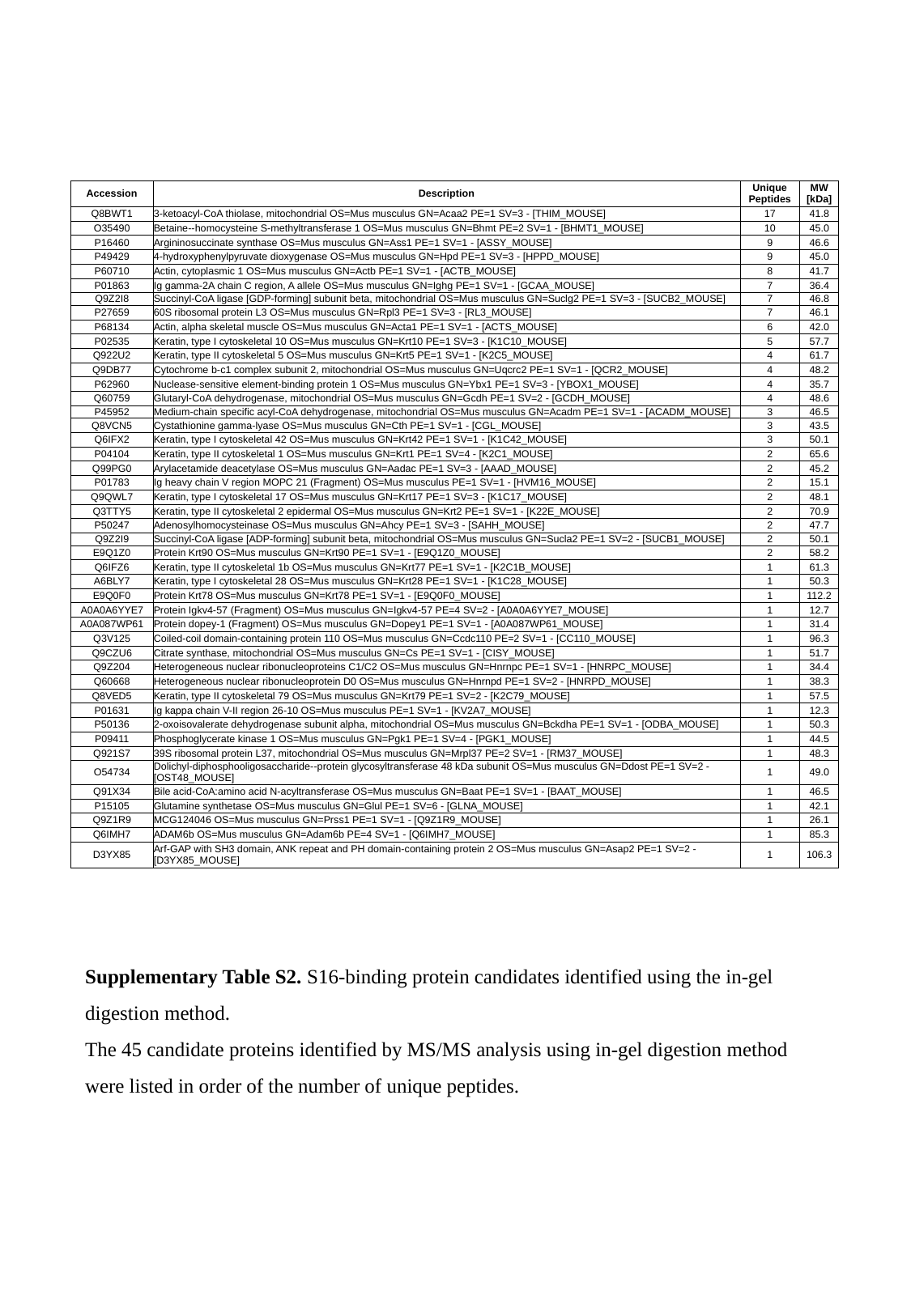

| Accession | Description | Unique Peptides | MW [kDa] |
| --- | --- | --- | --- |
| Q8BWT1 | 3-ketoacyl-CoA thiolase, mitochondrial OS=Mus musculus GN=Acaa2 PE=1 SV=3 - [THIM\_MOUSE] | 17 | 41.8 |
| O35490 | Betaine--homocysteine S-methyltransferase 1 OS=Mus musculus GN=Bhmt PE=2 SV=1 - [BHMT1\_MOUSE] | 10 | 45.0 |
| P16460 | Argininosuccinate synthase OS=Mus musculus GN=Ass1 PE=1 SV=1 - [ASSY\_MOUSE] | 9 | 46.6 |
| P49429 | 4-hydroxyphenylpyruvate dioxygenase OS=Mus musculus GN=Hpd PE=1 SV=3 - [HPPD\_MOUSE] | 9 | 45.0 |
| P60710 | Actin, cytoplasmic 1 OS=Mus musculus GN=Actb PE=1 SV=1 - [ACTB\_MOUSE] | 8 | 41.7 |
| P01863 | Ig gamma-2A chain C region, A allele OS=Mus musculus GN=Ighg PE=1 SV=1 - [GCAA\_MOUSE] | 7 | 36.4 |
| Q9Z2I8 | Succinyl-CoA ligase [GDP-forming] subunit beta, mitochondrial OS=Mus musculus GN=Suclg2 PE=1 SV=3 - [SUCB2\_MOUSE] | 7 | 46.8 |
| P27659 | 60S ribosomal protein L3 OS=Mus musculus GN=Rpl3 PE=1 SV=3 - [RL3\_MOUSE] | 7 | 46.1 |
| P68134 | Actin, alpha skeletal muscle OS=Mus musculus GN=Acta1 PE=1 SV=1 - [ACTS\_MOUSE] | 6 | 42.0 |
| P02535 | Keratin, type I cytoskeletal 10 OS=Mus musculus GN=Krt10 PE=1 SV=3 - [K1C10\_MOUSE] | 5 | 57.7 |
| Q922U2 | Keratin, type II cytoskeletal 5 OS=Mus musculus GN=Krt5 PE=1 SV=1 - [K2C5\_MOUSE] | 4 | 61.7 |
| Q9DB77 | Cytochrome b-c1 complex subunit 2, mitochondrial OS=Mus musculus GN=Uqcrc2 PE=1 SV=1 - [QCR2\_MOUSE] | 4 | 48.2 |
| P62960 | Nuclease-sensitive element-binding protein 1 OS=Mus musculus GN=Ybx1 PE=1 SV=3 - [YBOX1\_MOUSE] | 4 | 35.7 |
| Q60759 | Glutaryl-CoA dehydrogenase, mitochondrial OS=Mus musculus GN=Gcdh PE=1 SV=2 - [GCDH\_MOUSE] | 4 | 48.6 |
| P45952 | Medium-chain specific acyl-CoA dehydrogenase, mitochondrial OS=Mus musculus GN=Acadm PE=1 SV=1 - [ACADM\_MOUSE] | 3 | 46.5 |
| Q8VCN5 | Cystathionine gamma-lyase OS=Mus musculus GN=Cth PE=1 SV=1 - [CGL\_MOUSE] | 3 | 43.5 |
| Q6IFX2 | Keratin, type I cytoskeletal 42 OS=Mus musculus GN=Krt42 PE=1 SV=1 - [K1C42\_MOUSE] | 3 | 50.1 |
| P04104 | Keratin, type II cytoskeletal 1 OS=Mus musculus GN=Krt1 PE=1 SV=4 - [K2C1\_MOUSE] | 2 | 65.6 |
| Q99PG0 | Arylacetamide deacetylase OS=Mus musculus GN=Aadac PE=1 SV=3 - [AAAD\_MOUSE] | 2 | 45.2 |
| P01783 | Ig heavy chain V region MOPC 21 (Fragment) OS=Mus musculus PE=1 SV=1 - [HVM16\_MOUSE] | 2 | 15.1 |
| Q9QWL7 | Keratin, type I cytoskeletal 17 OS=Mus musculus GN=Krt17 PE=1 SV=3 - [K1C17\_MOUSE] | 2 | 48.1 |
| Q3TTY5 | Keratin, type II cytoskeletal 2 epidermal OS=Mus musculus GN=Krt2 PE=1 SV=1 - [K22E\_MOUSE] | 2 | 70.9 |
| P50247 | Adenosylhomocysteinase OS=Mus musculus GN=Ahcy PE=1 SV=3 - [SAHH\_MOUSE] | 2 | 47.7 |
| Q9Z2I9 | Succinyl-CoA ligase [ADP-forming] subunit beta, mitochondrial OS=Mus musculus GN=Sucla2 PE=1 SV=2 - [SUCB1\_MOUSE] | 2 | 50.1 |
| E9Q1Z0 | Protein Krt90 OS=Mus musculus GN=Krt90 PE=1 SV=1 - [E9Q1Z0\_MOUSE] | 2 | 58.2 |
| Q6IFZ6 | Keratin, type II cytoskeletal 1b OS=Mus musculus GN=Krt77 PE=1 SV=1 - [K2C1B\_MOUSE] | 1 | 61.3 |
| A6BLY7 | Keratin, type I cytoskeletal 28 OS=Mus musculus GN=Krt28 PE=1 SV=1 - [K1C28\_MOUSE] | 1 | 50.3 |
| E9Q0F0 | Protein Krt78 OS=Mus musculus GN=Krt78 PE=1 SV=1 - [E9Q0F0\_MOUSE] | 1 | 112.2 |
| A0A0A6YYE7 | Protein Igkv4-57 (Fragment) OS=Mus musculus GN=Igkv4-57 PE=4 SV=2 - [A0A0A6YYE7\_MOUSE] | 1 | 12.7 |
| A0A087WP61 | Protein dopey-1 (Fragment) OS=Mus musculus GN=Dopey1 PE=1 SV=1 - [A0A087WP61\_MOUSE] | 1 | 31.4 |
| Q3V125 | Coiled-coil domain-containing protein 110 OS=Mus musculus GN=Ccdc110 PE=2 SV=1 - [CC110\_MOUSE] | 1 | 96.3 |
| Q9CZU6 | Citrate synthase, mitochondrial OS=Mus musculus GN=Cs PE=1 SV=1 - [CISY\_MOUSE] | 1 | 51.7 |
| Q9Z204 | Heterogeneous nuclear ribonucleoproteins C1/C2 OS=Mus musculus GN=Hnrnpc PE=1 SV=1 - [HNRPC\_MOUSE] | 1 | 34.4 |
| Q60668 | Heterogeneous nuclear ribonucleoprotein D0 OS=Mus musculus GN=Hnrnpd PE=1 SV=2 - [HNRPD\_MOUSE] | 1 | 38.3 |
| Q8VED5 | Keratin, type II cytoskeletal 79 OS=Mus musculus GN=Krt79 PE=1 SV=2 - [K2C79\_MOUSE] | 1 | 57.5 |
| P01631 | Ig kappa chain V-II region 26-10 OS=Mus musculus PE=1 SV=1 - [KV2A7\_MOUSE] | 1 | 12.3 |
| P50136 | 2-oxoisovalerate dehydrogenase subunit alpha, mitochondrial OS=Mus musculus GN=Bckdha PE=1 SV=1 - [ODBA\_MOUSE] | 1 | 50.3 |
| P09411 | Phosphoglycerate kinase 1 OS=Mus musculus GN=Pgk1 PE=1 SV=4 - [PGK1\_MOUSE] | 1 | 44.5 |
| Q921S7 | 39S ribosomal protein L37, mitochondrial OS=Mus musculus GN=Mrpl37 PE=2 SV=1 - [RM37\_MOUSE] | 1 | 48.3 |
| O54734 | Dolichyl-diphosphooligosaccharide--protein glycosyltransferase 48 kDa subunit OS=Mus musculus GN=Ddost PE=1 SV=2 - [OST48\_MOUSE] | 1 | 49.0 |
| Q91X34 | Bile acid-CoA:amino acid N-acyltransferase OS=Mus musculus GN=Baat PE=1 SV=1 - [BAAT\_MOUSE] | 1 | 46.5 |
| P15105 | Glutamine synthetase OS=Mus musculus GN=Glul PE=1 SV=6 - [GLNA\_MOUSE] | 1 | 42.1 |
| Q9Z1R9 | MCG124046 OS=Mus musculus GN=Prss1 PE=1 SV=1 - [Q9Z1R9\_MOUSE] | 1 | 26.1 |
| Q6IMH7 | ADAM6b OS=Mus musculus GN=Adam6b PE=4 SV=1 - [Q6IMH7\_MOUSE] | 1 | 85.3 |
| D3YX85 | Arf-GAP with SH3 domain, ANK repeat and PH domain-containing protein 2 OS=Mus musculus GN=Asap2 PE=1 SV=2 - [D3YX85\_MOUSE] | 1 | 106.3 |
Supplementary Table S2. S16-binding protein candidates identified using the in-gel digestion method.
The 45 candidate proteins identified by MS/MS analysis using in-gel digestion method were listed in order of the number of unique peptides.

## Slide 19
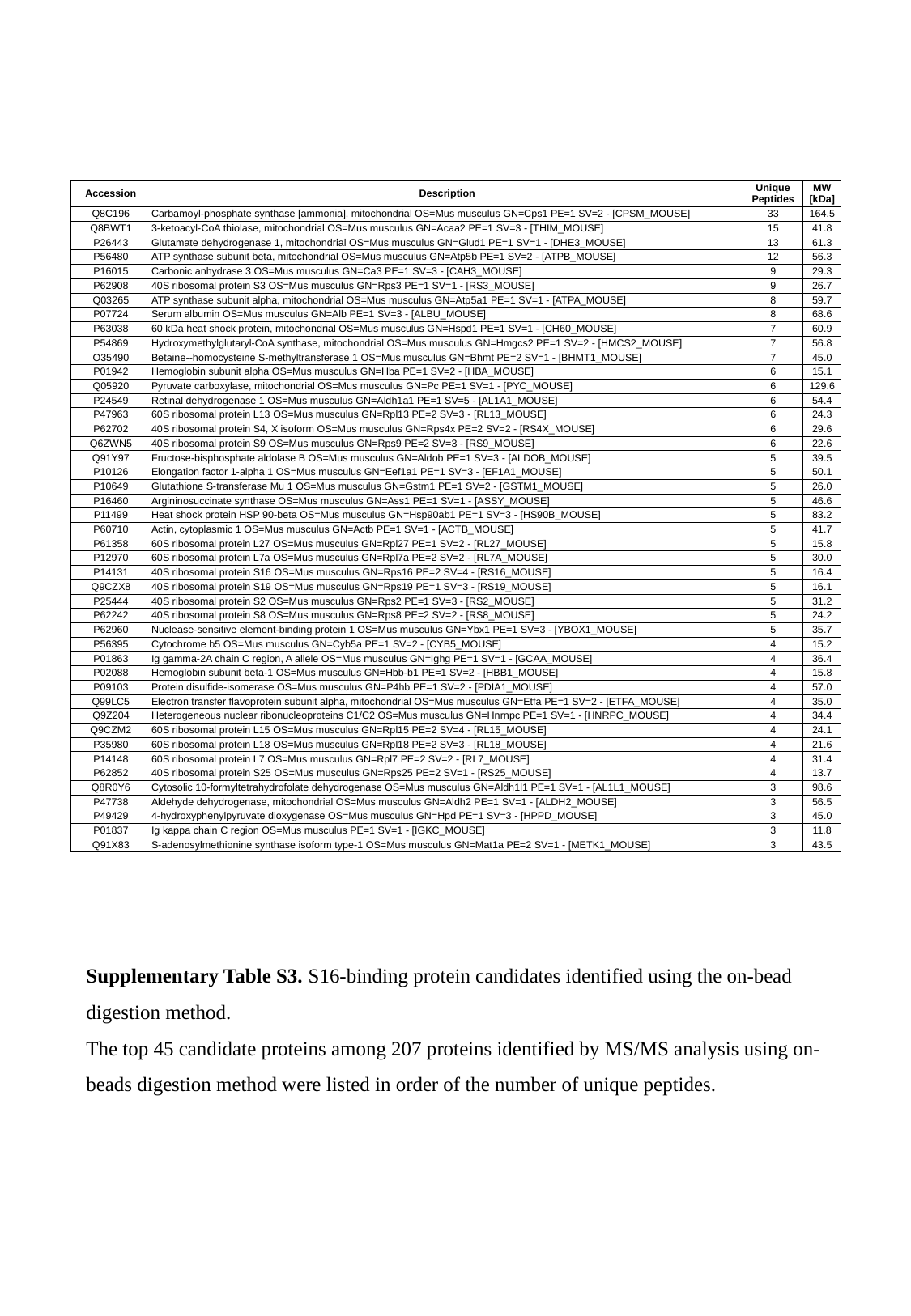

| Accession | Description | Unique Peptides | MW [kDa] |
| --- | --- | --- | --- |
| Q8C196 | Carbamoyl-phosphate synthase [ammonia], mitochondrial OS=Mus musculus GN=Cps1 PE=1 SV=2 - [CPSM\_MOUSE] | 33 | 164.5 |
| Q8BWT1 | 3-ketoacyl-CoA thiolase, mitochondrial OS=Mus musculus GN=Acaa2 PE=1 SV=3 - [THIM\_MOUSE] | 15 | 41.8 |
| P26443 | Glutamate dehydrogenase 1, mitochondrial OS=Mus musculus GN=Glud1 PE=1 SV=1 - [DHE3\_MOUSE] | 13 | 61.3 |
| P56480 | ATP synthase subunit beta, mitochondrial OS=Mus musculus GN=Atp5b PE=1 SV=2 - [ATPB\_MOUSE] | 12 | 56.3 |
| P16015 | Carbonic anhydrase 3 OS=Mus musculus GN=Ca3 PE=1 SV=3 - [CAH3\_MOUSE] | 9 | 29.3 |
| P62908 | 40S ribosomal protein S3 OS=Mus musculus GN=Rps3 PE=1 SV=1 - [RS3\_MOUSE] | 9 | 26.7 |
| Q03265 | ATP synthase subunit alpha, mitochondrial OS=Mus musculus GN=Atp5a1 PE=1 SV=1 - [ATPA\_MOUSE] | 8 | 59.7 |
| P07724 | Serum albumin OS=Mus musculus GN=Alb PE=1 SV=3 - [ALBU\_MOUSE] | 8 | 68.6 |
| P63038 | 60 kDa heat shock protein, mitochondrial OS=Mus musculus GN=Hspd1 PE=1 SV=1 - [CH60\_MOUSE] | 7 | 60.9 |
| P54869 | Hydroxymethylglutaryl-CoA synthase, mitochondrial OS=Mus musculus GN=Hmgcs2 PE=1 SV=2 - [HMCS2\_MOUSE] | 7 | 56.8 |
| O35490 | Betaine--homocysteine S-methyltransferase 1 OS=Mus musculus GN=Bhmt PE=2 SV=1 - [BHMT1\_MOUSE] | 7 | 45.0 |
| P01942 | Hemoglobin subunit alpha OS=Mus musculus GN=Hba PE=1 SV=2 - [HBA\_MOUSE] | 6 | 15.1 |
| Q05920 | Pyruvate carboxylase, mitochondrial OS=Mus musculus GN=Pc PE=1 SV=1 - [PYC\_MOUSE] | 6 | 129.6 |
| P24549 | Retinal dehydrogenase 1 OS=Mus musculus GN=Aldh1a1 PE=1 SV=5 - [AL1A1\_MOUSE] | 6 | 54.4 |
| P47963 | 60S ribosomal protein L13 OS=Mus musculus GN=Rpl13 PE=2 SV=3 - [RL13\_MOUSE] | 6 | 24.3 |
| P62702 | 40S ribosomal protein S4, X isoform OS=Mus musculus GN=Rps4x PE=2 SV=2 - [RS4X\_MOUSE] | 6 | 29.6 |
| Q6ZWN5 | 40S ribosomal protein S9 OS=Mus musculus GN=Rps9 PE=2 SV=3 - [RS9\_MOUSE] | 6 | 22.6 |
| Q91Y97 | Fructose-bisphosphate aldolase B OS=Mus musculus GN=Aldob PE=1 SV=3 - [ALDOB\_MOUSE] | 5 | 39.5 |
| P10126 | Elongation factor 1-alpha 1 OS=Mus musculus GN=Eef1a1 PE=1 SV=3 - [EF1A1\_MOUSE] | 5 | 50.1 |
| P10649 | Glutathione S-transferase Mu 1 OS=Mus musculus GN=Gstm1 PE=1 SV=2 - [GSTM1\_MOUSE] | 5 | 26.0 |
| P16460 | Argininosuccinate synthase OS=Mus musculus GN=Ass1 PE=1 SV=1 - [ASSY\_MOUSE] | 5 | 46.6 |
| P11499 | Heat shock protein HSP 90-beta OS=Mus musculus GN=Hsp90ab1 PE=1 SV=3 - [HS90B\_MOUSE] | 5 | 83.2 |
| P60710 | Actin, cytoplasmic 1 OS=Mus musculus GN=Actb PE=1 SV=1 - [ACTB\_MOUSE] | 5 | 41.7 |
| P61358 | 60S ribosomal protein L27 OS=Mus musculus GN=Rpl27 PE=1 SV=2 - [RL27\_MOUSE] | 5 | 15.8 |
| P12970 | 60S ribosomal protein L7a OS=Mus musculus GN=Rpl7a PE=2 SV=2 - [RL7A\_MOUSE] | 5 | 30.0 |
| P14131 | 40S ribosomal protein S16 OS=Mus musculus GN=Rps16 PE=2 SV=4 - [RS16\_MOUSE] | 5 | 16.4 |
| Q9CZX8 | 40S ribosomal protein S19 OS=Mus musculus GN=Rps19 PE=1 SV=3 - [RS19\_MOUSE] | 5 | 16.1 |
| P25444 | 40S ribosomal protein S2 OS=Mus musculus GN=Rps2 PE=1 SV=3 - [RS2\_MOUSE] | 5 | 31.2 |
| P62242 | 40S ribosomal protein S8 OS=Mus musculus GN=Rps8 PE=2 SV=2 - [RS8\_MOUSE] | 5 | 24.2 |
| P62960 | Nuclease-sensitive element-binding protein 1 OS=Mus musculus GN=Ybx1 PE=1 SV=3 - [YBOX1\_MOUSE] | 5 | 35.7 |
| P56395 | Cytochrome b5 OS=Mus musculus GN=Cyb5a PE=1 SV=2 - [CYB5\_MOUSE] | 4 | 15.2 |
| P01863 | Ig gamma-2A chain C region, A allele OS=Mus musculus GN=Ighg PE=1 SV=1 - [GCAA\_MOUSE] | 4 | 36.4 |
| P02088 | Hemoglobin subunit beta-1 OS=Mus musculus GN=Hbb-b1 PE=1 SV=2 - [HBB1\_MOUSE] | 4 | 15.8 |
| P09103 | Protein disulfide-isomerase OS=Mus musculus GN=P4hb PE=1 SV=2 - [PDIA1\_MOUSE] | 4 | 57.0 |
| Q99LC5 | Electron transfer flavoprotein subunit alpha, mitochondrial OS=Mus musculus GN=Etfa PE=1 SV=2 - [ETFA\_MOUSE] | 4 | 35.0 |
| Q9Z204 | Heterogeneous nuclear ribonucleoproteins C1/C2 OS=Mus musculus GN=Hnrnpc PE=1 SV=1 - [HNRPC\_MOUSE] | 4 | 34.4 |
| Q9CZM2 | 60S ribosomal protein L15 OS=Mus musculus GN=Rpl15 PE=2 SV=4 - [RL15\_MOUSE] | 4 | 24.1 |
| P35980 | 60S ribosomal protein L18 OS=Mus musculus GN=Rpl18 PE=2 SV=3 - [RL18\_MOUSE] | 4 | 21.6 |
| P14148 | 60S ribosomal protein L7 OS=Mus musculus GN=Rpl7 PE=2 SV=2 - [RL7\_MOUSE] | 4 | 31.4 |
| P62852 | 40S ribosomal protein S25 OS=Mus musculus GN=Rps25 PE=2 SV=1 - [RS25\_MOUSE] | 4 | 13.7 |
| Q8R0Y6 | Cytosolic 10-formyltetrahydrofolate dehydrogenase OS=Mus musculus GN=Aldh1l1 PE=1 SV=1 - [AL1L1\_MOUSE] | 3 | 98.6 |
| P47738 | Aldehyde dehydrogenase, mitochondrial OS=Mus musculus GN=Aldh2 PE=1 SV=1 - [ALDH2\_MOUSE] | 3 | 56.5 |
| P49429 | 4-hydroxyphenylpyruvate dioxygenase OS=Mus musculus GN=Hpd PE=1 SV=3 - [HPPD\_MOUSE] | 3 | 45.0 |
| P01837 | Ig kappa chain C region OS=Mus musculus PE=1 SV=1 - [IGKC\_MOUSE] | 3 | 11.8 |
| Q91X83 | S-adenosylmethionine synthase isoform type-1 OS=Mus musculus GN=Mat1a PE=2 SV=1 - [METK1\_MOUSE] | 3 | 43.5 |
Supplementary Table S3. S16-binding protein candidates identified using the on-bead digestion method.
The top 45 candidate proteins among 207 proteins identified by MS/MS analysis using on-beads digestion method were listed in order of the number of unique peptides.
